# Evaluation of Different Standard Amino Acids to Enhance the Biomass, Lipid, Fatty Acid, and γ-Linolenic Acid Production in *Rhizomucor pusillus* and *Mucor circinelloides*

**DOI:** 10.3389/fnut.2022.876817

**Published:** 2022-05-03

**Authors:** Hassan Mohamed, Mohamed F. Awad, Aabid Manzoor Shah, Yusuf Nazir, Tahira Naz, Abdallah Hassane, Shaista Nosheen, Yuanda Song

**Affiliations:** ^1^Colin Ratledge Center for Microbial Lipids, School of Agricultural Engineering and Food Science, Shandong University of Technology, Zibo, China; ^2^Department of Botany and Microbiology, Faculty of Science, Al-Azhar University, Assiut, Egypt; ^3^Department of Biology, College of Science, Taif University, Taif, Saudi Arabia; ^4^Department of Food Sciences, Faculty of Science and Technology, Universiti Kebangsaan Malaysia, Bangi, Malaysia

**Keywords:** lipid accumulation, fatty acids, GLA, nitrogen source, abiotic factors, *Rhizomucor pusillus*, *Mucor circinelloides*

## Abstract

In this study, 18 standard amino acids were tested as a single nitrogen source on biomass, total lipid, total fatty acid (TFA) production, and yield of γ-linolenic acid (GLA) in *Rhizomucor pusillus* AUMC 11616.A and *Mucor circinelloides* AUMC 6696.A isolated from unusual habitats. Grown for 4 days at 28°C, shaking at 150 rpm, the maximum fungal biomass for AUMC 6696.A was 14.6 ± 0.2 g/L with arginine and 13.68 ± 0.1 g/L with asparagine, when these amino acids were used as single nitrogen sources, while AUMC 11616.A maximum biomass was 10.73 ± 0.8 g/L with glycine and 9.44 ± 0.6 g/L with valine. These were significantly higher than the ammonium nitrate control (*p* < 0.05). The highest levels of TFA were achieved with glycine for AUMC 11616.A, 26.2 ± 0.8% w/w of cell dry weight, and glutamic acid for AUMC 6696.A, 23.1 ± 1.3%. The highest GLA yield was seen with proline for AUMC 11616.A, 13.4 ± 0.6% w/w of TFA, and tryptophan for AUMC 6696.A, 12.8 ± 0.3%, which were 38% and 25% higher than the ammonium tartrate control. The effects of environmental factors such as temperature, pH, fermentation time, and agitation speed on biomass, total lipids, TFA, and GLA concentration of the target strains have also been investigated. Our results demonstrated that nitrogen assimilation through amino acid metabolism, as well as the use of glucose as a carbon source and abiotic factors, are integral to increasing the oleaginicity of tested strains. Few studies have addressed the role of amino acids in fermentation media, and this study sheds light on *R. pusillus* and *M. circinelloides* as promising candidates for the potential applications of amino acids as nitrogen sources in the production of lipids.

## Introduction

Microorganisms known for naturally synthesizing lipids for more than 20% of their cell dry weight (CDW) are termed oleaginous microorganisms (1, 2). They have frequently been found to produce oils with compositional similarities to plants and fish (3). Nutritionally important polyunsaturated fatty acids (PUFAs), such as γ-linolenic acid (GLA), have been shown in various studies to be beneficial in the avoidance and treatment of multiple diseases (4, 5). Microorganisms have several advantages over traditional plants for producing GLA-rich oils, including simple cultural conditions, a high growth rate, and high yields that are not affected by changes in climatic conditions (6). Thus, GLA production by oleaginous microorganisms is a promising alternative to plant-based production. *Mucor circinelloides*, an oleaginous fungus, has been broadly employed to study GLA production, and it has been chosen as a model oleaginous microbe to produce GLA in various studies (7–11).

Nitrogen sources are essential components for microbial growth media, and several studies have demonstrated that nitrogen sources play important roles in growth conditions and bioactive compound production (12–14). The influences of nitrogen sources on lipid accumulation and unsaturated fatty acid production in oleaginous microorganisms, mainly fungi, have been extensively studied. Sodium nitrate stimulated cell growth, while also increasing lipid accumulation in *Neochloris oleoabundans* (15). Due to their effects on mycelial morphology, *Mortierella alpina* accumulated two times as much arachidonic acid when soybean meal was employed as a nitrogen source in the medium in the presence of yeast extract (16, 17). Organic nitrogen compounds are more favorable for cell growth and lipid production in oleaginous fungus *M. alpina* than inorganic nitrogen sources (18), and it has been observed that different amino acids, used as nitrogen supplementation, can differentially regulate gene expression, which in turn affects lipid production in *Saccharomyces cerevisiae* (19). However, few studies have been carried out to investigate the individual effects of each of the 18 amino acids on lipid biosynthesis in various oleaginous microorganisms.

Different strategies have been suggested for the development of lipid production in microorganisms. Among them, nitrogen restriction has been widely stated as an effective approach for the overproduction of storage lipids in oleaginous microbes (20). Recently, some reports have indicated that nitrogen limitation could also enhance lipid accumulation in oleaginous yeasts (21, 22). However, the impact of different nitrogen deficiency strategies on lipid production in oleaginous microbes, including yeast and filamentous fungi, is thus far unreported. In fermentation media, the carbon/nitrogen (C/N) ratio controls the switch between protein and lipid synthesis; therefore, the C/N ratio of culture media is one of the most critical nutritional factors affecting the total amount of produced lipids. Moreover, it has been observed that the initial C/N ratio should be higher than 20 for maximum lipid accumulation by oleaginous organisms (23, 24).

In our recent work, we have investigated multiple Mucoromycota fungi for GLA, lipid, and carotenoids content, and two strains, *Rhizomucor pusillus* AUMC 11616.A and *Mucor circinelloides* AUMC 6696.A, were chosen for this study, based on their high lipid content, as promising strains for lipid production (25). A literature survey on the oleaginous fungus *R. pusillus* did not reveal any studies on its nitrogen assimilation and lipid production. Therefore, the effects of 18 standard amino acids as single nitrogen sources on fungal growth, glucose consumption, lipid accumulation, and GLA content in *R. pusillus*, as well as in *M. circinelloides*, were investigated in this study. In this study, we explore the significance of using amino acids as nitrogen sources for improving the biotechnological application of oleaginous fungi for lipid accumulation.

## Materials and Methods

### Microorganisms

The strains *R. pusillus* AUMC 11616.A and *M. circinelloides* AUMC 6696.A were obtained from our previous work (25) as pure stock cultures of the laboratory of Microbial Lipids and Fermentation, School of Agriculture Engineering and Food Science, Shandong University of Technology. These strains were initially isolated from unusual niches (animal manure and textile [100%] polyester) and are characterized by the production of high lipid contents. The cultures were stored at 4–6°C on potato dextrose agar (PDA) slants (Hopebio Laboratories, China) and subcultured every 2–3 months.

### Microbiological Media and Culture Conditions

The Kendrick and Ratledge medium (K&R), which is rich in carbon and limited in nitrogen, was used in this study during all fermentation stages (26). In total, 100 μl spore suspensions of approximately 1 × 10^7^ spores/ml of *R. pusillus* or *M. circinelloides* were obtained from 5-day-old PDA cultures and inoculated separately into 500 ml baffled flasks containing 150 ml of K&R medium, which contained 30.0 g/L glucose, 3.30 g/L ammonium tartrate, 2.0 g/L Na_2_HPO_4_, 7.0 g/L KH_2_PO_4_, 1.50 g/L yeast extract, 1.50 g/L gSO_4_⋅7H_2_O, 0.1 g/L CaCl_2_⋅2H_2_O, 1.0 mg/L ZnSO_4_⋅7H_2_O, 8.0 mg/L FeCl_3_⋅6H_2_O, 0.1 mg/L Co(NO_3_)_2_⋅6H_2_O, 0.1 mg/L LMnSO_4_⋅5H_2_O, and 0.1 mg/L CuSO_4_⋅5H_2_O in 1,000 ml deionized H_2_O. All components were sterilized at 121°C for 20 min, except for glucose, which was added after sterilization. The cultures were incubated at 28°C with an agitation speed of 150 rpm and then used as a seed culture at 10% (v/v) to inoculate 500 ml baffled flasks with 150 ml modified K&R (N-limited medium), containing 80.0 g/L glucose and 0.5 g/L nitrogen, without yeast extract. Ammonium tartrate (control) and 18 amino acids were used as single nitrogen sources, and each medium contained 0.5 g nitrogen/L (e.g., 3.29 g/L ammonium tartrate, or 3.19 g/L alanine, or 2.68 g/L glycine, equivalent to 0.5 g/L nitrogen). The cultures were incubated for 96 h at 28°C with agitation at 150 rpm.

### Determination of Biomass Concentration

The fungal dry cell mass was harvested by filtration through a Buchner funnel under reduced pressure. The resulting fungal biomass was rinsed thrice with sterile deionized water to eliminate possible medium residues. After filtration, all samples were frozen overnight in a freeze-dryer at -80°C, lyophilized for a further 48 h, and then the fungal cell dry weight (CDW) was measured by the weighing method. This measurement was carried out in triplicate.

### Determination of Glucose Concentrations

Glucose concentration in the culture was measured using a biosensor analyzer (SBA-40E, Shandong University of Technology, China) according to the manufacturer’s instructions.

### Extraction of Total Lipids

Total lipids were extracted from the dried biomass according to the Folch method with some minor modifications (10). In brief, approximately 20 mg of fungal biomass was vigorously homogenized with 2 ml of 5 M HCl in lipid tubes. Then, these were placed in a water bath at 80°C for 4–5 h (vortexed every 1 h). The mixture was cooled down to room temperature and then added with 2 ml chloroform, 1 ml methanol, and 100 μl pentadecanoic acid as an internal standard (15:0 from Millipore, Sigma-Aldrich, United States), and the resulting mixture was vortexed for 30 s. For proper mixing, tubes were placed in a vertical 360 tube rotator for 1 h and then centrifuged at 3,000 rpm for 5 min to separate the two phases. The lower phase of 2 ml chloroform with extracted lipids was removed and transferred to a new tube. The solvent phase was evaporated under a stream of N_2_ and the total lipids were determined as% wt/wt of biomass.

### Analysis of Fatty Acid Profile

Fatty acid methyl esters (FAME) were extracted by initially adding 1 ml of 10% methanol in HCl at 60°C for 3 h, followed by 2 ml *n*-Hexane and 1 ml saturated NaCl. All tubes were vortexed for 30–60 s, placed in a vertical 360 tube rotator for 1 h, and then centrifuged at 3,000 rpm for 5 min. The resultant FAMEs were subsequently analyzed by gas chromatography (GC). The GC machine was equipped with a flame ionization detector (FID) and the capillary column DB-FFAP (30 m × 320 μm × 0.25 μm film thickness: Agilent Scientific Instruments), with the temperature program increasing from 160 to 230°C at 8°C min. Following the analysis, a ballistic increase to 300°C allows cleaning of the column during a hold of 2 min. The detector gases were air and hydrogen; their flow rates were regulated at 400 and 30 ml/min, respectively. Nitrogen was used as a make-up gas at 25 ml/min. The injection volume was 1 μl with a total run time of 25 min. Identification and quantification of individual chromatographic peaks were carried out by comparison to the external fatty acid methyl ester standard mixture (Supelco^®^ 37 Component FAME Mix).

### Effect of Various Environmental Factors on Tested Strains

Based on the results showing high lipid content, asparagine and glutamine amino acids were chosen as nitrogen sources to investigate the maximum biomass, lipid, fatty acids, and GLA content of *R. pusillus* AUMC 11616.A and *M. circinelloides* AUMC 6696.A, respectively, under selected various abiotic factors. Notably, 150 ml of K&R fermentation medium in 500 ml flasks with asparagine or glutamine was used in this experiment. Four different abiotic factors were used to test their effect on fungal biomass, total lipids, TFA, and GLA yields of the tested strains. To investigate the effects of pH, it was adjusted for values of 4–8. The temperature varied in the range of 15 ± 2°C–40 ± 2°C, and agitation speed ranged from 100 to 250 rpm, while different incubation periods of 1–6 days were also studied.

### Statistical Analysis

All data of the experiments were statistically analyzed with three replicates, and the results were presented as mean ± SD (*n* = 3). Student’s *t*-test was performed in SPSS 16.0 for statistical analysis of the data, and *p* < 0.05 was considered significantly different.

## Results

### Cultural Characteristics and Phylogenetic Tree Construction

**FIGURE 1:**
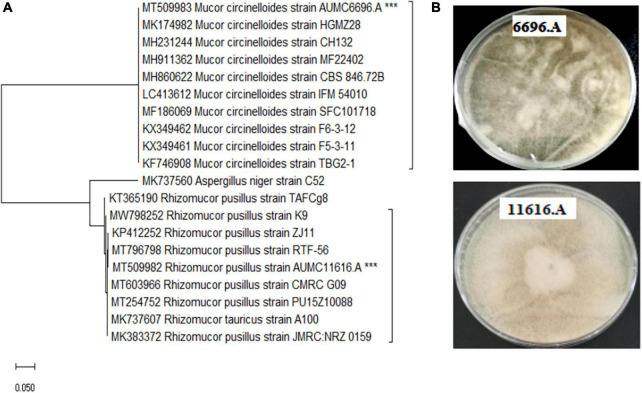
**(A)** The neighbor-joining (NJ) phylogenetic tree based on ITS gene sequences of AUMC 6696.A and AUMC 11616.A, with closely related strains accessed from the GenBank using BLASTN (http://www.ncbi.nlm.nih.gov/blast/). These sequences were aligned using ClustalW. Bootstrap values included 500 replicates for the NJ method using MEGA software (version 11.0.6). ***indicates tested strain. **(B)** Morphological characteristics of tested strains on agar plates.

The selected strains *R. pusillus* AUMC 11616.A and *M. circinelloides* AUMC 6696.A were pre-identified based on their morphology and confirmed by the ITS-5.8S ribosomal gene sequences method. The target strains were characterized as fast-growing Mucorales, producing white to gray colonies on PDA agar plates. The ITS ribosomal gene sequence results of AUMC 11616.A and AUMC 6696.A were correlated with the phylogenetic position as shown in Figure 1. The microscopic examinations of these strains are shown in Supplementary Figure 1.

### Effects of 18 Amino Acids on the Growth and Glucose Utilization of Tested Strains

**FIGURE 2:**
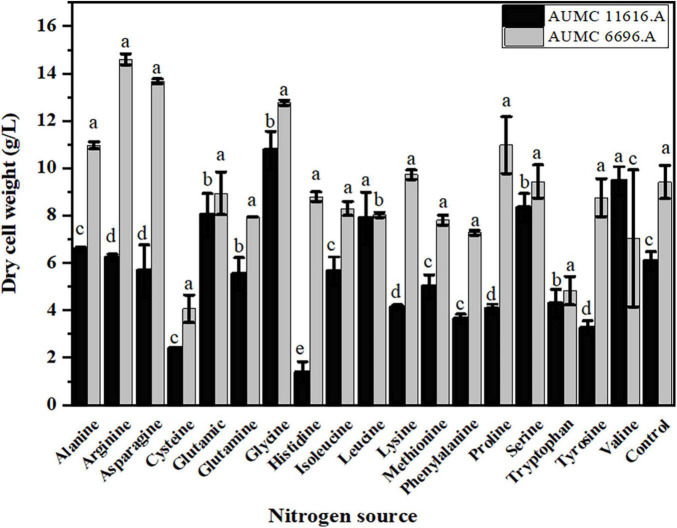
Biomass concentration (g/L) of *R. pusillus* AUMC 11616.A and *M. circinelloides* AUMC 6696.A strains grown at 30°C for 4 days, on different nitrogen sources. Ammonium tartrate was employed as the control. Error bars represent the average standard deviation from three biological replicates. Values that show different superscripts were significantly different from each other using *t*-test (*p* < 0.05).

The selected fungal strains AUMC 11616.A and AUMC 6696.A were cultivated in the K&R fermentation media for 4 days. Fungal biomass concentration with different nitrogen sources was determined (Figure 2). When compared to ammonium tartrate as control, alanine, proline, glycine, especially arginine and asparagine, stimulated the growth of AUMC 6696.A, and glutamic acid, serine, leucine, especially valine and glycine, stimulated the growth of AUMC 11616.A. When arginine and asparagine were used as single nitrogen sources by AUMC 6696.A, the CDW reached up to 14.5 and 13.6 g/L, respectively, which were 35% and 30% higher than that of ammonium tartrate. In the case of AUMC 11616.A with valine and glycine as nitrogen sources, the CDW reached up to 9.4 and 10.8 g/L, respectively, which were 36% and 42% higher than the control. Contrastingly, the fungal biomass production of the selected strains grown on cysteine, tryptophan, valine, and histidine for AUMC 6696.A, or cysteine and tyrosine for AUMC 11616.A, were markedly lower than that of the ammonium tartrate control. Our findings suggested that these amino acids were not good nitrogen sources for the optimal growth of these fungi. These results showed varied significant impacts on biomass dry weight when compared with their control, which may affect lipid metabolism and further impact fatty acid biosynthesis and GLA in AUMC 6696.A and AUMC 11616.A.

**FIGURE 3:**
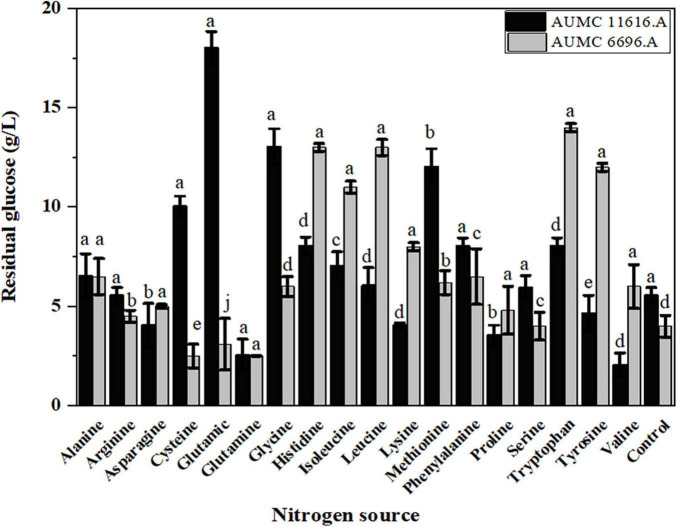
The residual glucose concentration (g/L) of *R. pusillus* AUMC 11616.A and *M. circinelloides* AUMC 6696.A strains grown at 30°C for 4 days, on different nitrogen sources. Ammonium tartrate was employed as the control. Error bars represent the average standard deviation from three biological replicates. Values that show different superscripts were significantly different from each other using *t*-test (*p* < 0.05).

Since glucose is utilized as a carbon source during fungal growth, the residual glucose concentration was determined after 4 days of fermentation, showing that the rate of glucose consumption was different in these two strains, dependent on the amino acid source. As shown in Figure 3, the residual glucose concentrations of AUMC 11616.A grown on glutamic acid, glycine, methionine, or cysteine and AUMC 6696.A grown on tryptophan, tyrosine, leucine or histidine were significantly higher than that of ammonium tartrate (initial value 80 g/L), and the fungal biomass production was correspondingly lower than that of the control, as shown in Figure 2. While the residual glucose concentrations of AUMC 6696.A grown on cysteine, glutamic acid, and glutamine were markedly lower than that of the control, AUMC 11616.A grown on glutamine, proline, and valine were determined to have the lowest concentrations of residual glucose.

### Influence of Amino Acids on Total Lipids, Fatty Acids, and Their Composition, and GLA Content in Selected Strains

**FIGURE 4:**
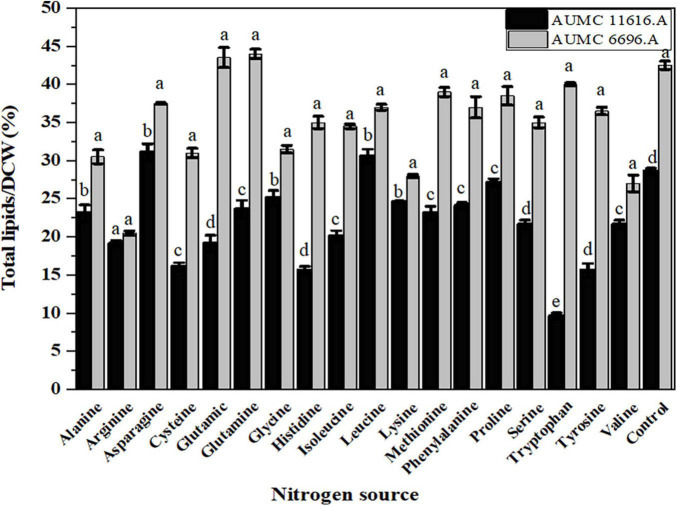
Total lipids production of *R. pusillus* AUMC 11616.A and *M. circinelloides* AUMC 6696.A strains grown at 30°C for 4 days, on different nitrogen sources. Ammonium tartrate was employed as the control. Error bars represent the average standard deviation from three biological replicates. Values that show different superscripts were significantly different from each other using *t*-test (*p* < 0.05).

To investigate the effect of different amino acids on lipid accumulation in AUMC 6696.A and AUMC 11616.A, lipid analysis of these fungi was performed. The resulting CDWs of the tested strains after fermentation were evaluated for their total lipid content and fatty acid composition. Based on our results, the AUMC 6696.A produced higher lipid content than AUMC 11616.A during the cultivation of all tested amino acids. Data in Figure 4 show the lipid content of the AUMC 11616.A grown on asparagine (31 ± 1.1) and leucine (30.5 ± 09) and AUMC 6696.A grown on glutamine (44.5 ± 1.3) and glutamic acid (43.5 ± 0.2%) had the greatest lipid production. Most amino acid sources, including arginine, glycine, lysine, valine, serine, and cysteine, decreased lipid production in both tested strains compared with the control, while proline and alanine, for AUMC 11616.A, and tryptophan and methionine, for AUMC 6696.A, had no significant effect (*p* < 0.001) on lipid production. These results suggest that asparagine and leucine for AUMC 11616.A and glutamine and glutamic acid for AUMC6696.A were the optimal nitrogen sources for total lipid accumulation. Among tested amino acids, the tested strain AUMC6696.A exhibited not much significant difference in lipid content when compared to control, while asparagine and leucine in AUMC 11616.A strain showed significantly higher in their lipid content when compared with ammonium tartrate as a control.

**FIGURE 5:**
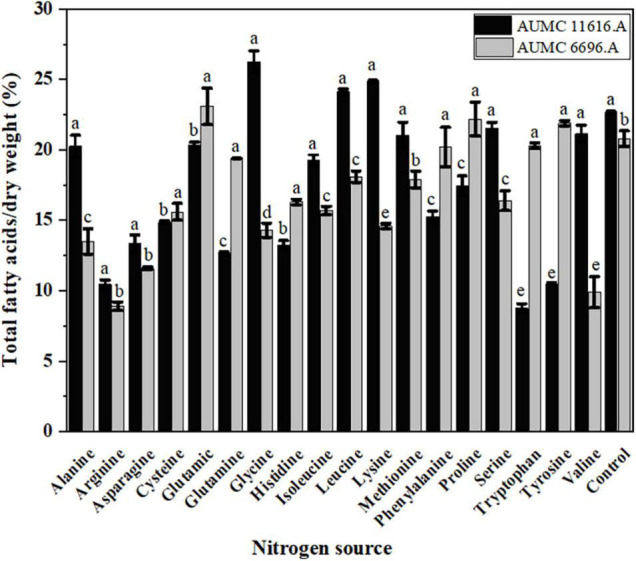
Total fatty acids (TFAs) production of *R. pusillus* AUMC 11616.A and *M. circinelloides* AUMC 6696.A strains grown at 30°C for 4 days, on different nitrogen sources. Ammonium tartrate was employed as the control. Error bars represent the average standard deviation from three biological replicates. Values that show different superscripts were significantly different from each other using *t*-test (*p* < 0.05).

Total fatty acid production of AUMC 11616.A grown on glycine and AUMC 6696.A grown on glutamic acid were 26.2 ± 0.8 and 23.1 ± 1.3% (w/w) of CDW, respectively, which were the highest among tested nitrogen sources and 1.19 and 1.11 times that of ammonium tartrate controls (Figure 5). Notably, tryptophan in AUMC 11616.A (8.9 ± 0.3) and arginine in AUMC 6696.A (8.7 ± 0.2%) produced the lowest TFAs. These findings indicated no significant impact (*p* < 0.061) on TFAs in both tested strains compared to control.

To investigate the effects of 18 amino acids on the TFA composition of AUMC 11616.A and AUMC 6696.A, strains were grown on glucose as carbon source and with one of the 18 amino acids as nitrogen source, with the same C/N ratio and with the same culture conditions. Oleic acid (C18:1) was the predominant fatty acid grown with each amino acid in both tested strains. Of the 18 amino acids, glutamic acid for AUMC 11616.A and serine for AUMC 6696.A prominently enhanced the stearic acid (18:0) concentration within TFAs 2.17- and 3.9-fold, respectively, compared to ammonium tartrate controls. Additionally, tyrosine for AUMC 11616.A and valine for AUMC 6696.A also greatly improved the palmitoleic acid (C16:1) concentration of TFAs 3.2- and 2.3-fold compared to controls, respectively. A significant decrease in GLA concentration was seen for AUMC 11616.A grown on phenylalanine (74% of control), tryptophan (57%), and histidine (49%), as well as for AUMC 6696.A grown on asparagine (16.5%), leucine (15.1%), and cysteine (13.9%), compared to controls. A potential explanation for this could be due to the relevant desaturases (i.e., Delta-6-desaturase) for the synthesis of GLA being suppressed when these fungi were grown on certain amino acids. Although proline and tryptophan strongly increased the GLA concentration of the TFAs by 25% and 38% in their respective strains, overall fungal biomass and lipid production were not produced in sufficient quantities, therefore showing these are not suitable nitrogen sources for GLA production. Detailed FAs chromatographic analyses based on GC results are listed in Supplementary Figure 2, and FAs profile of these fungi are shown in Table 1. Through comprehensive analysis, the

**TABLE 1:** Fatty acid composition of *R. pusillus* AUMC 11616.A and *M. circinelloides* AUMC 6696.A fungi grown on different amino acids at 28°C for 4 days.

Amino acids (N-source)	Composition of fatty acid (% TFA)													
	C14:0		C16:0		C16:1		C18:0		C18:1		C18:2		C18:3	
	11616.A	6696.A	11616.A	6696.A	11616.A	6696.A	11616.A	6696.A	11616.A	6696.A	11616.A	6696.A	11616.A	6696.A
Alanine	2.2 ± 0.1	3.5 ± 0.1	27.3 ± 0.2	21.1 ± 0.4	1.4 ± 0.1	5.2 ± 0.1	12.6 ± 0.1	3.5 ± 0.1	35.7 ± 1.3	48.7 ± 2.1	12.7 ± 0.2	10.5 ± 0.2	8.1 ± 0.1	7.5 ± 0.1
Arginine	4.3 ± 0.1	3.7 ± 0.1	22.5 ± 0.3	16.8 ± 0.1	8.1 ± 0.1	6.2 ± 0.1	2.3 ± 0.1	3.1 ± 0.13	43.2 ± 1.2	42.5 ± 1.3	13.5 ± 0.2	15.1 ± 0.2	5.9 ± 0.1	12.6 ± 0.1
Asparagine	4.5 ± 0.1	3.0 ± 0.0	20.0 ± 1.2	19.3 ± 1.1	5.1 ± 0.2	4.0 ± 0.1	4.7 ± 0.3	3.0 ± 0.1	37.6 ± 0.6	52.8 ± 1.3	17.6 ± 0.8	10.8 ± 0.1	10.7 ± 0.1	6.6 ± 0.2
Cysteine	4.5 ± 0.1	3.5 ± 0.1	26.3 ± 0.8	23.7 ± 0.5	3.3 ± 0.1	3.1 ± 0.1	9.0 ± 0.2	4.1 ± 0.1	32.5 ± 1.4	50.5 ± 1.9	13.0 ± 0.2	8.7 ± 0.2	11.5 ± 0.2	6.8 ± 0.1
Glutamic acid	0.8 ± 0.0	2.5 ± 0.1	27.5 ± 0.6	21.7 ± 0.4	0.9 ± 0.1	3.4 ± 0.1	18.4 ± 0.4	4.1 ± 0.1	33.4 ± 0.9	45.4 ± 1.8	12.7 ± 0.2	10.9 ± 0.1	6.6 ± 0.1	12.1 ± 0.2
Glutamine	3.5 ± 0.1	2.5 ± 0.1	17.6 ± 0.2	21.7 ± 0.3	6.1 ± 0.1	2.8 ± 0.1	2.1 ± 0.1	4.1 ± 0.1	37.5 ± 03	46.4 ± 0.5	21.2 ± 0.2	11.8 ± 0.1	12.1 ± 0.1	10.5 ± 0.1
Glycine	0.7 ± 0.0	3.1 ± 0.1	27.2 ± 0.8	16.6 ± 0.7	1.2 ± 0.0	4.1 ± 0.1	16.6 ± 0.2	3.8 ± 0.1	33.8 ± 1.4	47.9 ± 1.1	14.0 ± 0.3	12.6 ± 0.1	6.9 ± 0.1	12.0 ± 0.1
Histidine	4.7 ± 0.1	2.1 ± 0.1	30.2 ± 0.8	24.9 ± 1.3	2.7 ± 0.1	1.8 ± 0.1	17.4 ± 0.6	6.2 ± 0.1	29.3 ± 1.5	48.4 ± 1.8	10.8 ± 0.2	8.6 ± 0.1	5.1 ± 0.1	8.2 ± 0.1
Isoleucine	2.1 ± 0.0	3.2 ± 0.1	29.3 ± 0.5	19.6 ± 0.4	1.6 ± 0.0	3.7 ± 0.1	11.5 ± 0.1	3.0 ± 0.1	34.5 ± 0.6	46.7 ± 1.2	15.2 ± 0.8	12.9 ± 0.6	7.5 ± 0.1	11.0 ± 0.2
Leucine	1.8 ± 0.1	3.0 ± 0.1	28.3 ± 0.4	24.7 ± 0.6	1.4 ± 0.0	3.7 ± 0.1	14.7 ± 0.1	4.5 ± 0.1	36.3 ± 1.2	47.0 ± 1.4	12.2 ± 0.3	10.4 ± 0.2	5.5 ± 0.1	6.7 ± 0.1
Lysine	2.2 ± 0.0	3.0 ± 0.0	31.0 ± 0.8	25.7 ± 0.4	2.4 ± 0.0	2.0 ± 0.0	8.2 ± 0.4	9.2 ± 0.3	34.9 ± 0.9	36.7 ± 0.6	12.3 ± 0.2	11.7 ± 0.1	9.0 ± 0.1	11.9 ± 0.1
Methionine	3.0 ± 0.0	2.7 ± 0.1	22.0 ± 0.3	21.6 ± 0.4	3.8 ± 0.1	2.9 ± 0.1	4.6 ± 0.1	4.0 ± 0.1	44.7 ± 1.2	48.7 ± 1.3	12.9 ± 0.2	11.2 ± 0.1	9.1 ± 0.1	8.9 ± 0.1
Phenylalanine	0.2 ± 0.1	2.2 ± 0.1	25.1 ± 0.4	20.1 ± 0.1	1.8 ± 0.1	3.3 ± 0.1	9.3 ± 0.2	4.1 ± 0.13	47.8 ± 1.4	48.1 ± 1.2	11.8 ± 0.2	11.9 ± 0.1	2.3 ± 0.1	10.6 ± 0.1
Proline	4.3 ± 0.1	2.3 ± 0.1	19.5 ± 1.1	21.3 ± 0.8	4.8 ± 0.1	3.7 ± 0.2	2.9 ± 0.0	4.2 ± 0.1	39.0 ± 0.6	44.7 ± 1.1	16.4 ± 0.3	11.2 ± 0.2	13.4 ± 0.2	12.3 ± 0.1
Serine	2.4 ± 0.1	2.6 ± 0.1	24.7 ± 0.4	18.8 ± 0.2	1.1 ± 0.1	1.8 ± 0.1	14.2 ± 0.3	3.2 ± 0.2	31.3 ± 1.2	45.4 ± 1.7	12.7 ± 0.3	13.5 ± 0.2	6.1 ± 0.1	13.4 ± 0.1
Tryptophan	0.3 ± 0.0	1.7 ± 0.1	23.6 ± 0.3	18.3 ± 0.2	1.3 ± 0.0	2.6 ± 0.1	12.7 ± 0.1	3.7 ± 0.1	40.1 ± 1.2	48.6 ± 1.6	15.9 ± 0.5	12.5 ± 0.4	4.3 ± 0.1	12.8 ± 0.4
Tyrosine	6.8 ± 0.3	2.1 ± 0.1	22.8 ± 0.5	24.8 ± 0.6	8.8 ± 0.3	2.6 ± 0.1	8.1 ± 0.1	5.5 ± 0.1	33.6 ± 1.1	44.3 ± 1.2	11.9 ± 0.4	10.2 ± 0.2	8.2 ± 0.1	10.9 ± 0.5
Valine	0.2 ± 0.0	4.3 ± 0.1	28.5 ± 1.3	16.6 ± 0.8	0.9 ± 0.1	6.9 ± 0.1	15.1 ± 0.8	1.9 ± 0.8	34.2 ± 1.2	47.2 ± 1.3	15.3 ± 0.6	14.4 ± 1.1	5.2 ± 0.1	8.3 ± 0.1
Control	2.5 ± 0.0	2.8 ± 0.1	26.3 ± 0.4	23.0 ± 0.2	2.7 ± 0.1	3.0 ± 0.1	8.5 ± 0.1	4.1 ± 0.1	38.9 ± 0.9	48.1 ± 0.7	11.2 ± 0.2	11.1 ± 0.2	10.0 ± 0.1	7.9 ± 0.1

*Ammonium tartrate was employed as a control.*

FAs composition of the tested strains was significantly increased compared to the control.

**FIGURE 6:**
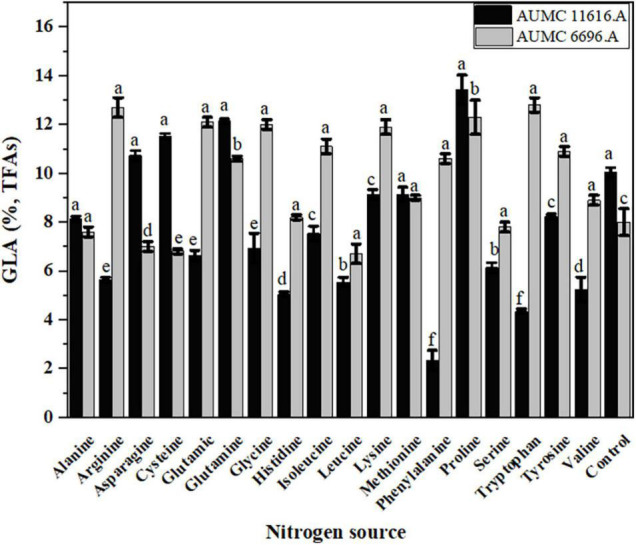
Effect of different amino acids on the GLA content of TFAs in *R. pusillus* AUMC 11616.A and *M. circinelloides* AUMC 6696.A strains grown at 30°C for 4 days, on different nitrogen sources. Ammonium tartrate was employed as the control. Error bars represent the average standard deviation from three biological replicates. Values that show different superscripts were significantly different from each other using *t*-test (*p* < 0.05).

**FIGURE 7:**
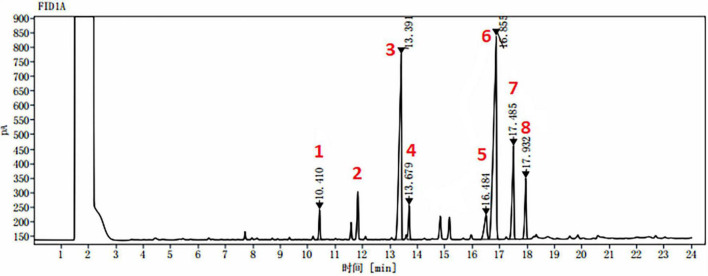
A typical GC chromatogram of target FAs identified from all tested samples. **1**. myristic acid “C14:0” at retention time: 10.410 min; **2**. pentadecanoic acid as standard “C15:0” at retention time: 11.910 min; **3**. palmitic acid “C16:0” at retention time: 13.391 min; **4**. palmitoleic acid “C16:1” at retention time: 13.679 min; **5**. stearic acid “C18:0” at retention time: 16.484 min; **6**. oleic acid “C18:1” at retention time: 16.855 min; **7**. linoleic acid “C18:2” at retention time: 17.485 min; and **8**. γ-linolenic acid “C18:3” at retention time: 17.932 min.

The effect of each amino acid as a nitrogen source on the GLA content of TFAs in these strains is shown in Figure 6. Most amino acids markedly decreased the GLA content of TFAs when compared to control, particularly in AUMC 11616.A. Some amino acids, such as lysine and methionine for AUMC 11616.A and alanine and histidine for AUMC 6696.A, had no prominent effect on the GLA content of TFAs, and only proline and tryptophan, as above, significantly increased the GLA content of TFAs in their respective strain. Peaks of different FAs in the tested samples were identified and confirmed by comparing with the retention time stability and peak area repeatability of the standard mixtures. Target FAs in all tested samples appeared relatively clear in GC chromatography as shown in Figure 7 (as an example).

### Effect of Abiotic Factors on AUMC11616.A and AUMC6696.A

K&R media containing asparagine for AUMC 11616.A and glutamine for AUMC 6696.A as nitrogen sources were employed to investigate the effects of abiotic factors, including fermentation time, pH, temperature, and agitation speed, on producing the greatest fungal biomass, total lipids, TFA, and GLA content with these strains. In this investigation, the tested strains were grown for 1, 2, 3, 4, 5, and 6 days. The highest GLA percentage of total fatty acids was 16 ± 0.6% for AUMC 11616.A and 15 ± 0.2% for AUMC 6696.A at the end of the 3rd day, and 19 ± 0.7 and 23 ± 0.9%, respectively, at the end of the 2nd day of incubation, while total lipids, as a percentage of CDW (46 ± 1.5% for AUMC 11616.A and 43 ± 1.2% for AUMC 6696.A) and biomass (12 ± 1.1 g/L for AUMC 11616.A and 11 ± 0.6 g/L for AUMC 6696.A) were seen to be highest at the end of the 4th, 5th, and 6th days of fermentation. Fermentation media with pH ranging from 4 to 8 were also tested. Cultures were grown with various pH levels at 28°C for 4 days. The greatest total lipids (45 ± 1.4% of CDW for AUMC 11616.A and 44 ± 0.8% for AUMC 6696.A) and biomass (10 ± 0.3 g/L for AUMC 11616.A and 10.5 ± 0.6 g/L for AUMC 6696.A) were seen at pH 6. Notably, total biomass and lipid production significantly dropped under suboptimal pH conditions. At this pH, AUMC 6696.A achieved the maximum TFA and GLA content of 23 ± 0.5% and 15 ± 0.8%, respectively. However, the maximum production of TFA and GLA for AUMC 11616.A was observed at pH 7 to 8 with 11 ± 0.7% and 13 ± 0.4%, respectively.

To determine the effect of temperature, tested strains were inoculated in fermentation media incubated at temperatures from 15°C to 40°C for 4 days. At 35°C, *R. pusillus* AUMC 11616.A produced maximum biomass (10 ± 0.4 g/L) and TFA content (22 ± 0.4%), while maximum total lipids (42 ± 1.0%) and GLA concentration (17 ± 1.5%) were seen at 30 and 20°C, respectively. *M. circinelloides* AUMC 6696.A produced the highest biomass (12 ± 0.4 g/L), TFA (25 ± 0.3%), and GLA contents (16 ± 0.2%) at 25°C and the highest total lipid content (42 ± 0.6%) at 35°C.

**FIGURE 8:**
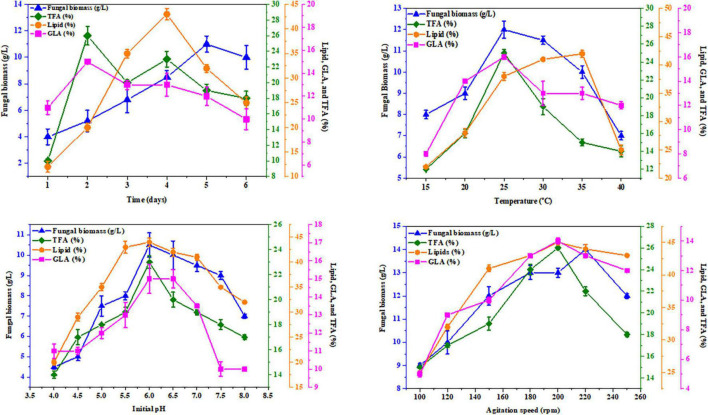
Effect of fermentation time, temperature, initial pH, and agitation speed on the biomass, total lipids, GLA content, and TFAs in *M. circinelloides* AUMC 6696.A. Glutamine was employed as the nitrogen source. Error bars represent the average standard deviation from three biological replicates.

**FIGURE 9:**
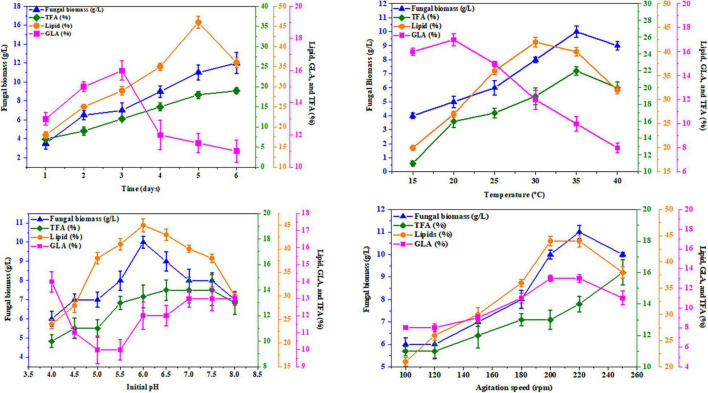
Effect of fermentation time, temperature, initial pH, and agitation speed on the biomass, total lipids, GLA content, and TFAs in *R. pusillus* AUMC 11616.A. Asparagine amino acid was employed as the nitrogen source. Error bars represent the average standard deviations of three biological replicates.

Finally, the agitation speed required for maximum total lipids (44 ± 0.1%) and GLA content (13 ± 0.3%) in AUMC 11616.A was observed at 200–240 rpm, with the highest biomass (11 ± 0.3 g/L) seen at 200 rpm and the highest TFA (16 ± 0.8%) seen at 240 rpm. The results for AUMC 6696.A showed the highest levels of total lipids (45 ± 0.4%), TFA (26 ± 0.2%), and GLA content (14 ± 0.2%) at 200 rpm, while biomass (14 ± 0.2 g/L) was greatest at 220 rpm. The results for all abiotic factors are illustrated in Figures 8, 9.

## Discussion

In this study, K&R medium supplemented with certain amino acids as single nitrogen sources was used to obtain higher biomass with a shorter fermentation period for cultures of *R. pusillus* AUMC 11616.A and *M. circinelloides* AUMC 6696.A, and this bioprocess revealed that some amino acids had obvious growth-promoting effects on these oleaginous strains. In previous studies, the ratio of C/N played an essential role in biomass growth and lipid accumulation (24, 27), and the accumulation of lipids by oleaginous fungi is triggered by the exhaustion of exogenous nitrogen in the culture medium (28, 29). These studies have shown that a low ratio of C/N in the early stage of fermentation was beneficial to the microorganism growth; however, a high C/N ratio could enhance the lipid accumulation of oleaginous microorganisms in the subsequent fermentation stage (1, 30). Additionally, amino acids were found to be ideal nitrogen sources as they are used to synthesize proteins, enzymes, and deoxyribonucleic acid, thus promoting growth.

When investigating the effects of different amino acids, under the same culture conditions, on the biomass of *M. circinelloides* AUMC 6696.A and *R. pusillus* AUMC 11616.A, we found significant enhancements in growth with certain amino acids, reaching up to 10.5 ± 0.1 g/L for AUMC 6696.A and 12.6 ± 0.1 g/L for AUMC 11616.A. In an earlier study by Lu et al. (18), multiple organic nitrogen molecules were more suitable for fungal cell growth and lipid production than inorganic nitrogen sources in *Mortierella alpina* (19). Oleaginous yeast *Rhodotorula glutinis* cultivated in a medium containing amino acids had significantly enhanced cell biomass at 13.63 g/L after 3 days compared with the basic medium as a control (31). In another fungus *Cunninghamella echinulata*, it was also found that glutamine and lysine as nitrogen sources produced poor cell growth compared with the ammonium salt (32), and tyrosine appeared to be the most favorable nitrogen source for the cell growth and TFA production in the oleaginous fungus *Mucor circinelloides* CBS108.16 (33). Furthermore, in a recent study by Shah et al. (34), they found that glycine and proline produced the highest biomass and total lipid content, compared to the ammonium tartrate control, in the endophytic oleaginous fungus *Gilbertella persicaria* DSR1 (34). In our work, the 18 standard amino acids differentially affected the growth of *M. circinelloides* AUMC 6696.A and *R. pusillus* AUMC 11616.A, with glycine appearing to be a more favorable nitrogen source for growth.

Several carbon substrates are important to microbial cell growth, and the process of lipid formation in the cell involves the metabolic conversion of external carbon sources into carbohydrates or hydrocarbons, subsequently resulting in lipid accumulation (3, 35). In this study, glucose was employed as the sole carbon substrate. Lipid accumulation in oleaginous microorganisms, particularly fungi, grown on the glucose was regulated by fatty acid *de novo* synthesis, which was not seen when grown on fatty substrates (36). Since fungal growth might be associated with the utilization of a simple carbon source such as glucose, the concentration of residual glucose was determined after 96 h of fermentation in our selected strains. As with our findings, oleaginous fungus *R. glutinis* showed higher utilization, and rapid consumption of glucose in the cultivation medium, when supplied with different amino acids, increased biomass by 0.32 g/L after 5 days (31).

Lipids are stored as droplets within the fungal cells, mainly in the form of triacylglycerol, and have been considered a valuable and renewable alternative source of high-value byproducts including PUFAs, and GLA in particular (37). Therefore, the studied strains were selected for their high lipid production. Based on our results, these strains produced higher total lipid content during cultivation with certain amino acids: asparagine and leucine for AUMC 11616.A and glutamine and glutamic acid for AUMC 6696.A. Glycine and glutamic also increased TFA content. Previous studies have shown that the type and composition of nitrogen sources and the initial C/N molar ratio in the medium influence lipid accumulation and fatty acid composition in oleaginous fungi, primarily reported in *Mucoromycota* (26, 34, 38). Total lipids, in the presence of ammonium bicarbonate, urea, tyrosine, tryptophan, phenylalanine, glutamine, glutamic acid, isoleucine, and histidine, ranged between 33 and 37% in oleaginous fungus *G. persicaria* DSR1, indicating that these nitrogen sources are suitable for lipid production (30). Our findings are almost consistent with those of Evans and Ratledge, where they showed that when glutamate, arginine, urea, or NH_4_Cl were used as nitrogen sources, lipid accumulation in *R. toruloides* CBS14 increased (39). Early reports on oleaginous fungi, such as *M. alpina*, have also shown that organic sources of nitrogen are more suitable for lipid production than inorganic sources (19). Moreover, research on *M. circinelloides* has shown that various amino acids used as nitrogen sources can influence cell biomass, fatty acids, and GLA yields (33). Among such microorganisms, oleaginous Zygomycetes are documented for their high oil production and for producing special fatty acids such as γ-linolenic acid (GLA), which other oleaginous microbes cannot synthesize in large quantities (26, 35, 40).

In this study, the GLA content of TFAs was significantly increased with certain amino acids, particularly with proline for AUMC 11616.A and tryptophan for AUMC 6696.A. In other studies, GLA has been shown to play an important role in humans, improving the health of patients suffering from diabetes, aging, cancer, and immune diseases (26, 41). The highest yield of GLA in this study was significantly more than that obtained from other species of Mucorales and some other Zygomycotina, which are considered potential candidates for the application in the industrial production of PUFAs and other valuable compounds (34). The recent advancement of GLA production in some selected oleaginous *Mucoromycota* is summarized in Table 2.

**TABLE 2:** GLA comparative analysis among various studies by selected *Mucromycota.*

Organism	C source	N source	GLA (%)	Reference
*Mucor fragilis* UBOCC-A-109196	Glucose	Yeast extract	24.5	(42)
*M. circinelloides* AUMC6027	Glucose	C_4_H_9_NO_6_ and yeast extract	10.65	(26)
*M. circinelloides* CBS 277.49	Glucose	Diammonium tartrate	27	(43)
*M. circinelloides* WJ11	Glucose	Diammonium tartrate	14.5	
*M. circinelloides* CBS 108.16	Glucose	Tyrosine	8	(33)
*M. hiemalis* AUMC6031	Glucose	C_4_H_9_NO_6_ and yeast extract	10.34	(26)
*Cunninghamella blakesleeana* JSK2	Glucose	KNO3	21	(44)
*C. echinulata* ATHUM 4411	Starch and xylose	NH_4_^+^ and yeast extract	5–11.2	(45, 46)
*Gilbertella persicaria* DSR1	Glucose	Proline and peptone	12.71	(34)
*Mort. isabellina* ATHUM 2935	Xylose, whey, and cheese lactose	NH_4_^+^, yeast extract, and cheese whey,	2.5–3	(46, 47)
*Rhizopus stolonifer* VKM F-400	Glucose	Yeast extract	20.3	(42)
*R. pusillus* AUMC 11616.A	Glucose	Proline	13.36	This study
*M. circinelloides* AUMC 6696.A	Glucose	Tryptophan	12.77	This study

There is a continuing interest in modifying the biochemical composition of fungal species by influencing abiotic or environmental factors. It is well studied that the chemical composition of microorganisms is affected by varied environmental conditions (48). Temperature plays a vital role in growth and lipid accumulation and can alter the composition of cellular fatty acids (34). It was observed that the GLA content of *Mucor rouxii* strain CFR-G15 increased significantly from 14.2% to 21.97% when the incubation temperature was lowered to 14°C (49). Our results demonstrate that the total lipid content, TFA, and GLA concentration decrease at 25 and 30°C as compared to the standard culturing temperature of 28°C. It is a well-known phenomenon that lower temperatures have a stimulatory effect on the biosynthesis of unsaturated fatty acids, which is considered as an adaptive mechanism in microbial cells to retain membrane fluidity (50, 51). It has also been reported that the interactions between pH values and components of cultivation media affect the ability of microorganisms to utilize the available nutrients in the medium (52). Moreover, earlier studies on oleaginous filamentous fungi reported an optimum incubation time of 4–5 days for high GLA and lipid yields (53). Therefore, all further fermentation experiments with the tested strains were incubated for 6 days. Depending on the microbe cultivated and the bio-product studied, increasing agitation speed has also been reported to reduce or promote productivity (54, 55). In another study by Saad et al., their experimental results revealed that lipid and GLA content were increased in samples agitated at 600 and 440 rpm, with values as high as 38.71% and 0.058 (g/g), respectively, in oleaginous fungus *Cunninghamella bainieri* 2A1 (53).

## Conclusion

This study showed that different amino acid nitrogen sources can affect the cell biomass, fatty acid profile, and yield of GLA in *Rhizomucor pusillus* AUMC 11616.A and *Mucor circinelloides* AUMC 6696.A, which are promising strains for the high production of total lipids and GLA. When cultivated for 4 days at 28°C, shaking at 150 rpm, arginine and asparagine, for AUMC 11616.A, and glycine and valine, for AUMC 6696.A, were the most favorable amino acid nitrogen sources for cell growth, while glycine (AUMC 11616.A) and glutamic acid (AUMC 6696.A) showed the highest TFA production. Additionally, the highest yield of GLA was seen when proline (AUMC 11616.A) and tryptophan (AUMC 6696.A) were employed as sole nitrogen sources. The influence of environmental factors such as temperature, pH, fermentation time, agitation speed on the biomass, total lipids, fatty acid, and GLA concentration of the target strains has been investigated, and the optimal conditions were identified. Based on the significant enhancement in their lipid production and PUFAs, the pentose phosphate pathway could be induced by certain studied amino acids, which increases the activity of lipogenic enzymes. This study serves as a basis for future bioprocess developments of therapeutically important high value-added products, such as GLA, as well as for using amino acids as nitrogen sources. Concerning research on amino acid assimilation, further work is needed to study the genes involved during fermentation and their biochemical pathways.

## Data Availability Statement

The original contributions presented in the study are included in the article/Supplementary Material, further inquiries can be directed to the corresponding authors.

## Author Contributions

HM and YS: conceptualization. HM and MA: methodology. HM and YN: software. HM, AH, and YN: validation. HM, MA, and TN: formal analysis. AS and AH: investigation. HM and SN: data curation. HM and AH: writing – original draft preparation. YS: writing – review and editing and supervision, project administration, and funding acquisition. TN and MA: visualization. All authors have read and agreed to the published version of the manuscript.

## Conflict of Interest

The authors declare that the research was conducted in the absence of any commercial or financial relationships that could be construed as a potential conflict of interest.

## Publisher’s Note

All claims expressed in this article are solely those of the authors and do not necessarily represent those of their affiliated organizations, or those of the publisher, the editors and the reviewers. Any product that may be evaluated in this article, or claim that may be made by its manufacturer, is not guaranteed or endorsed by the publisher.

## Supplementary Material

Supplementary Figure 1 Microscopic Images of lactophenol cotton blue-stained mycelia of *Rhizomucor pusillus* AUMC 11616.A **(A)** branched *sporangiophores* and **(B)** primitive *rhizoides*; and *M. circinelloides* AUMC 6696.A **(C)** *sporangiophores* and **(D)** *sporangiophores* carrying sporangium and spores.

Supplementary Figure 2 The GC results of the detailed FAs chromatographic analyses.
