# Supplementary material for: Evaluation of Different Standard Amino Acids to Enhance the Biomass, Lipid, Fatty Acid, and γ-Linolenic Acid Production in Rhizomucor pusillus and Mucor circinelloides
Source: Front Nutr. 2022 May 3;9:876817. doi: 10.3389/fnut.2022.876817 (PMC9112836; doi:10.3389/fnut.2022.876817)
Supplement: Supplementary Figure 1 — Microscopic Images of lactophenol cotton blue-stained mycelia of Rhizomucor pusillus AUMC 11616.A (A) branched sporangiophores and (B) primitive rhizoides; and M. circinelloides AUMC 6696.A (C) sporangiophores and (D) sporangiophores carrying sporangium and spores. [file Image_1.pdf]

## Supplementary Figure S1

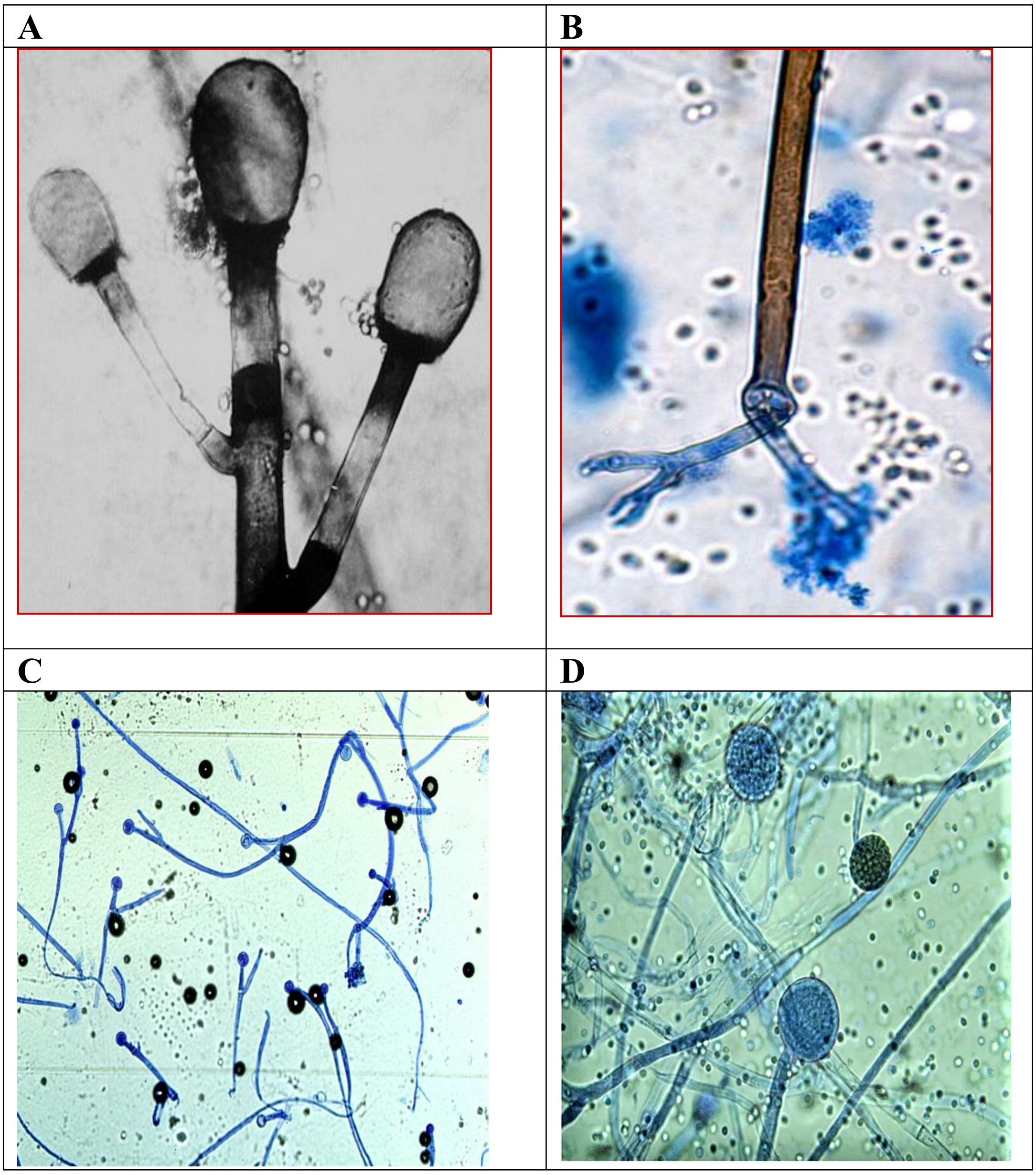


**Figure S1.** Microscopic Images of lactophenol cotton blue-stained mycelia of *Rhizomucor pusillus* AUMC 11616.A (A) branched sporangiophores and (B) primitive rhizoides; and *M. circinelloides* AUMC 6696.A (C) sporangiophores and (D) sporangiophores carrying sporangium and spores.
