# Supplementary material for: Evaluation of Different Standard Amino Acids to Enhance the Biomass, Lipid, Fatty Acid, and γ-Linolenic Acid Production in Rhizomucor pusillus and Mucor circinelloides
Source: Front Nutr. 2022 May 3;9:876817. doi: 10.3389/fnut.2022.876817 (PMC9112836; doi:10.3389/fnut.2022.876817)
Supplement: Supplementary Figure 2 — The GC results of the detailed FAs chromatographic analyses. [file Image_2.pdf]

单次进样报告

数据文件:	6696.A Serine20200610 085433.dx		
序列名称:	6696.A profile 1	项目名称:	脂肪酸
样品名称:	6696.A Serine	操作者:	系统
仪器:	7890B	进样日期:	2020-06-10 09:41:59+08:00
进样体积:	1.000	位置:	101
采集方法:	脂肪酸测定方法25min.amx	类型:	样品
处理方法:	GC_LC<br>面积百分比_DefaultMethod.pmx	样品含量:	0.00
手动修改:	手动积分		

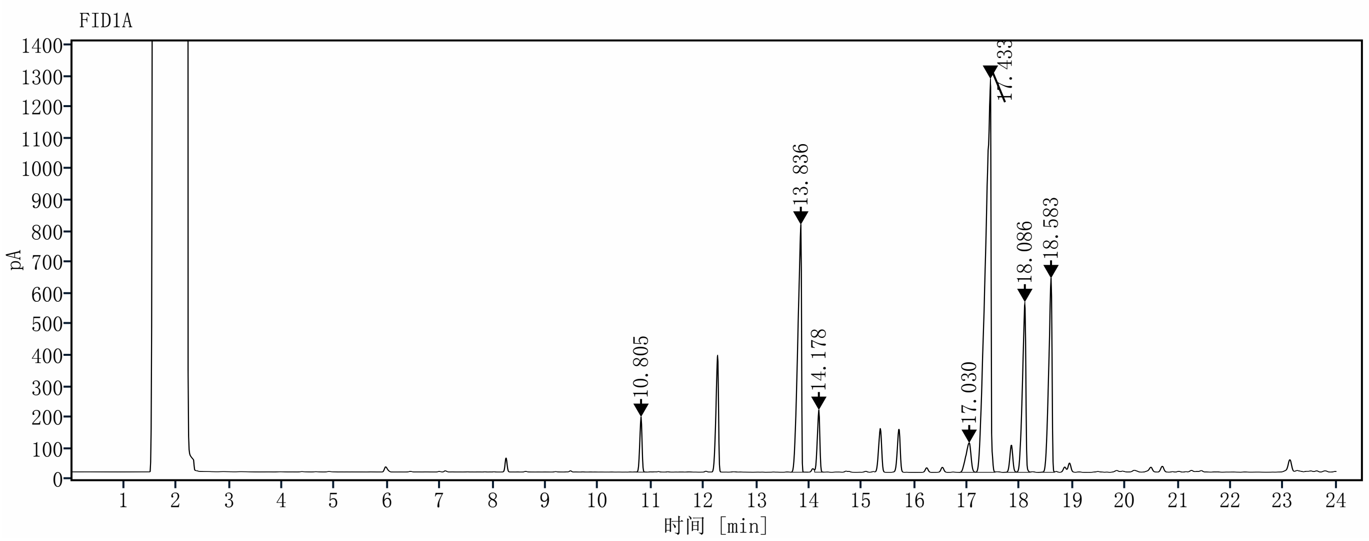

信号: FID1A

名称	化合物 浓度	化合物 含量	保留时间 [min]	类型	峰面积	峰面积%
			10.805	VB	525.76	2.57
			13.836	VB	3844.60	18.78
			14.178	VB	663.51	3.24
			17.030	BV	650.39	3.18
			17.433	VV	9289.88	45.38
			18.086	BV m	2758.76	13.48
			18.583	BV	2736.56	13.37
				总和	20469.46	

数据文件:	6696.A Alanine20200610 100601.dx		
序列名称:	6696.A profile 1	项目名称:	脂肪酸
样品名称:	6696.A Alanine	操作者:	系统
仪器:	7890B	进样日期:	2020-06-10 10:10:16+08:00
进样体积:	1.000	位置:	102
采集方法:	脂肪酸测定方法25min.amx	类型:	样品
处理方法:	GC_LC<br>面积百分比_DefaultMethod.pmx	样品含量:	0.00
手动修改:	手动积分		

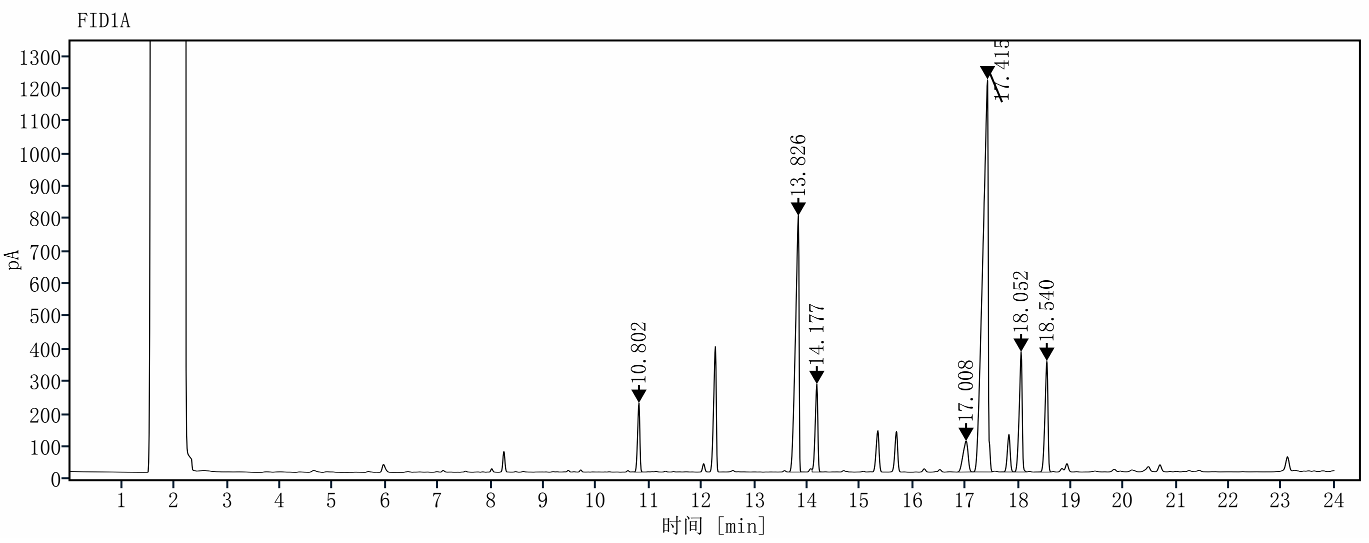

信号: FID1A

名称	化合物 浓度	化合物 含量	保留时间 [min]	类型	峰面积	峰面积%
			10.802	VB	629.61	3.54
			13.826	VB	3743.89	21.02
			14.177	VB	930.70	5.23
			17.008	BB	630.07	3.54
			17.415	BV	8662.55	48.64
			18.052	BV m	1872.10	10.51
			18.540	BV	1341.52	7.53
				总和	17810.44	

单次进样报告

Agilent Technologies

**数据文件:**	6696.A Arginine20200610 103418.dx		
**序列名称:**	6696.A profile 1	**项目名称:**	脂肪酸
**样品名称:**	6696.A Arginine	**操作者:**	系统
**仪器:**	7890B	**进样日期:**	2020-06-10 10:38:38+08:00
**进样体积:**	1.000	**位置:**	103
**采集方法:**	脂肪酸测定方法25min.amx	**类型:**	样品
**处理方法:**	GC_LC<br>面积百分比_DefaultMethod.pmx	**样品含量:**	0.00
**手动修改:**	手动积分		

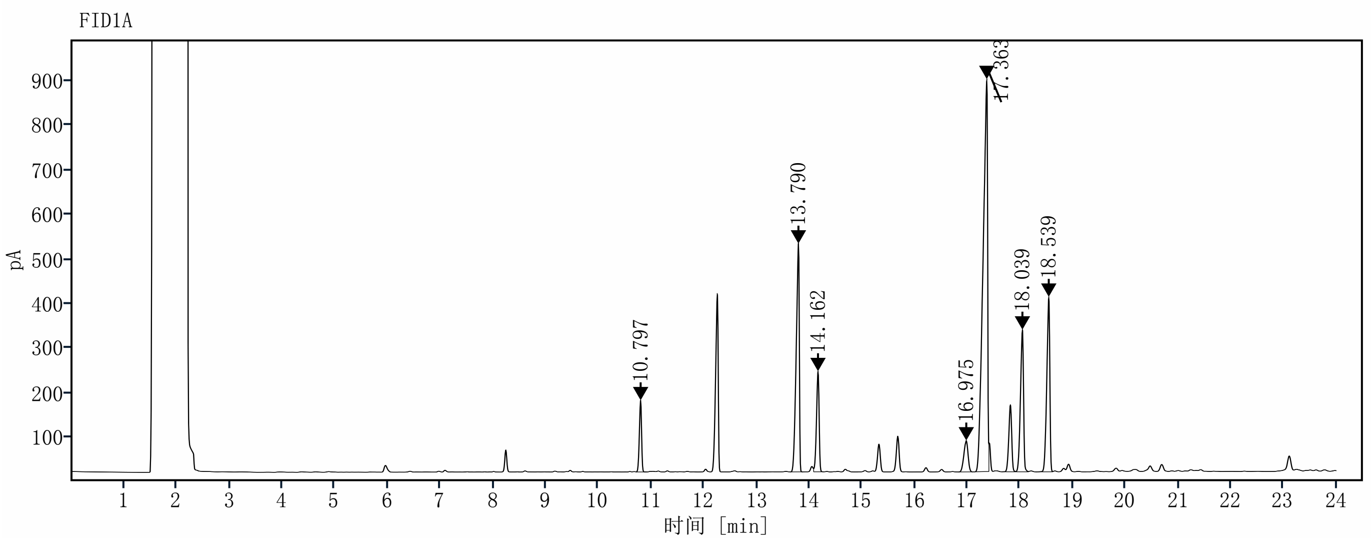

信号: FID1A

名称	化合物 浓度	化合物 含量	保留时间 [min]	类型	峰面积	峰面积%
			10.797	VB	468.66	3.78
			13.790	BB	2080.86	16.78
			14.162	VB	773.57	6.24
			16.975	BB	383.73	3.09
			17.363	BV	5267.19	42.47
			18.039	BV m	1864.46	15.03
			18.539	BV	1563.57	12.61
				总和	12402.04	

单次进样报告

**数据文件:**	6696.A Glutamine20200610 110239.dx		
**序列名称:**	6696.A profile 1	**项目名称:**	脂肪酸
**样品名称:**	6696.A Glutamine	**操作者:**	系统
**仪器:**	7890B	**进样日期:**	2020-06-10 11:07:06+08:00
**进样体积:**	1.000	**位置:**	104
**采集方法:**	脂肪酸测定方法25min.amx	**类型:**	样品
**处理方法:**	GC_LC<br>面积百分比_DefaultMethod.pmx	**样品含量:**	0.00
**手动修改:**	手动积分		

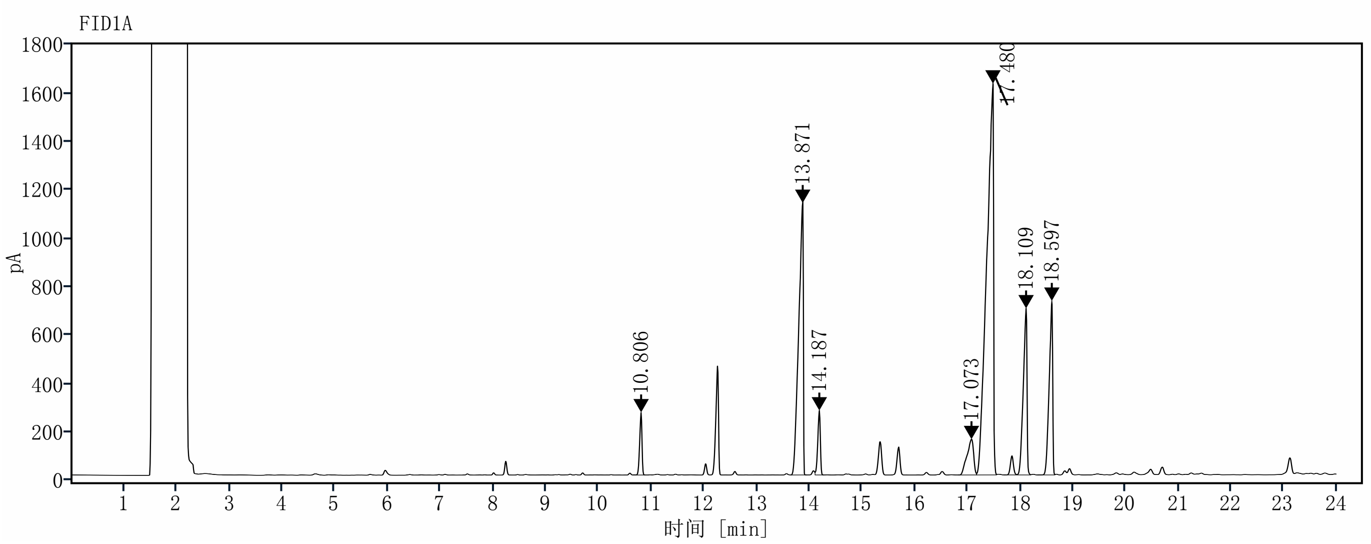

信号: FID1A

名称	化合物 浓度	化合物 含量	保留时间 [min]	类型	峰面积	峰面积%
			10.806	VB	781.17	2.52
			13.871	VB	6748.12	21.77
			14.187	VB	893.12	2.88
			17.073	BV	1283.56	4.14
			17.480	VV	14371.49	46.37
			18.109	BV m	3657.52	11.80
			18.597	BV	3257.59	10.51
				总和	30992.56	

单次进样报告

数据文件:	6696.A Tryptophane20200610 113107.dx		
序列名称:	6696.A profile 1	项目名称:	脂肪酸
样品名称:	6696.A Tryptophane	操作者:	系统
仪器:	7890B	进样日期:	2020-06-10 11:35:32+08:00
进样体积:	1.000	位置:	105
采集方法:	脂肪酸测定方法25min.amx	类型:	样品
处理方法:	GC_LC<br>面积百分比_DefaultMethod.pmx	样品含量:	0.00
手动修改:	手动积分		

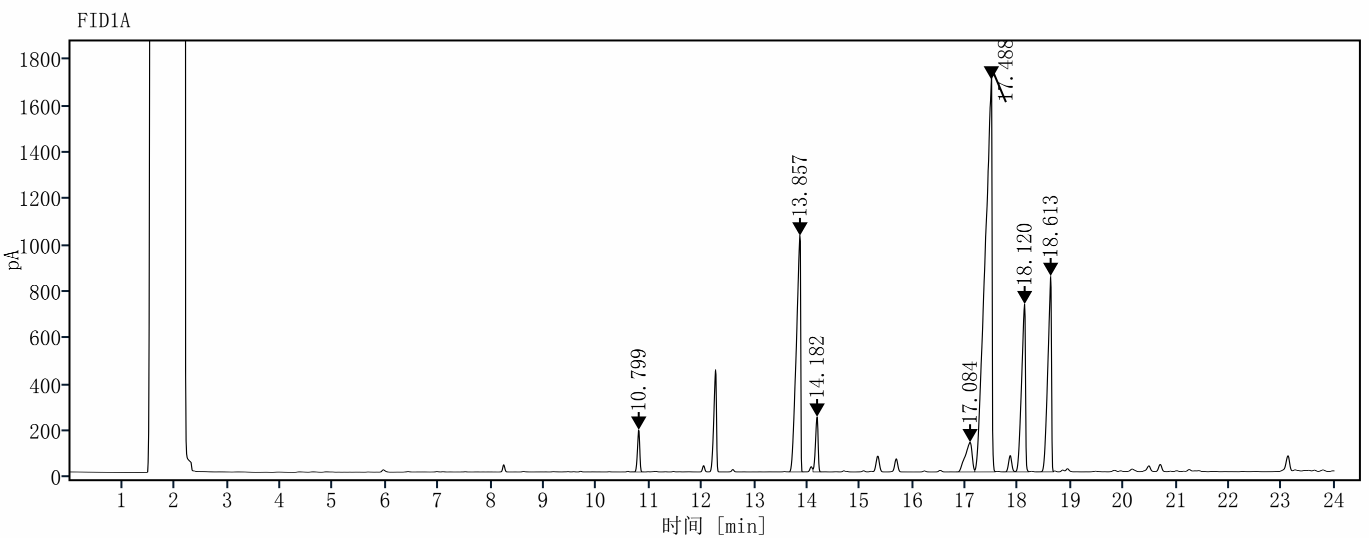

信号: FID1A

名称	化合物 浓度	化合物 含量	保留时间 [min]	类型	峰面积	峰面积%
			10.799	VB	545.01	1.72
			13.857	VV	5799.74	18.26
			14.182	VB	814.42	2.56
			17.084	BV	1156.66	3.64
			17.488	VV	15417.63	48.54
			18.120	BV m	3974.04	12.51
			18.613	BV	4057.13	12.77
				总和	31764.64	

单次进样报告

数据文件:	6696.A Tyrosine20200610 115934.dx		
序列名称:	6696.A profile 1	项目名称:	脂肪酸
样品名称:	6696.A Tyrosine	操作者:	系统
仪器:	7890B	进样日期:	2020-06-10 12:03:59+08:00
进样体积:	1.000	位置:	106
采集方法:	脂肪酸测定方法25min.amx	类型:	样品
处理方法:	GC_LC 面积百分比_DefaultMethod.pmx	样品含量:	0.00
手动修改:	手动积分		

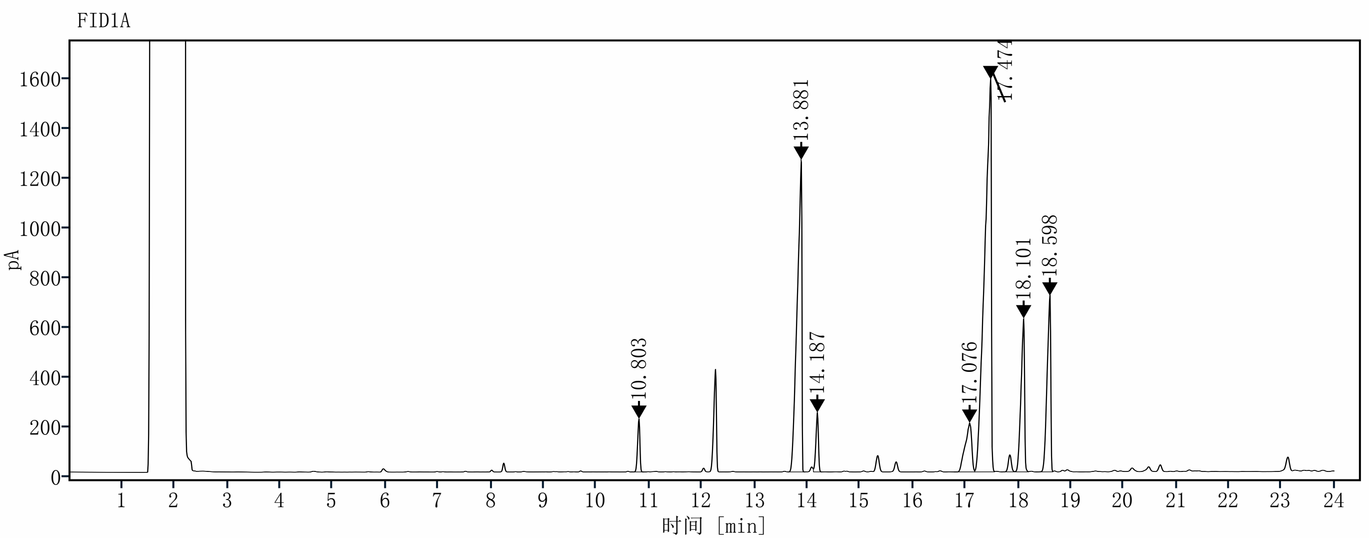

信号: FID1A

名称	化合物 浓度	化合物 含量	保留时间 [min]	类型	峰面积	峰面积%
			10.803	VB	650.87	2.10
			13.881	VV	7653.76	24.70
			14.187	VB	782.39	2.52
			17.076	BV	1678.63	5.42
			17.474	VV	13716.97	44.26
			18.101	BV m	3141.65	10.14
			18.598	BV	3365.07	10.86
				总和	30989.33	

单次进样报告

Agilent Technologies

数据文件: 6696.A Valine20200610 122801.dx

序列名称:	6696.A profile 1	项目名称:	脂肪酸
样品名称:	6696.A Valine	操作者:	系统
仪器:	7890B	进样日期:	2020-06-10 12:32:31+08:00
进样体积:	1.000	位置:	107
采集方法:	脂肪酸测定方法25min.amx	类型:	样品
处理方法:	GC_LC<br>面积百分比_DefaultMethod.pmx	样品含量:	0.00
手动修改:	手动积分		

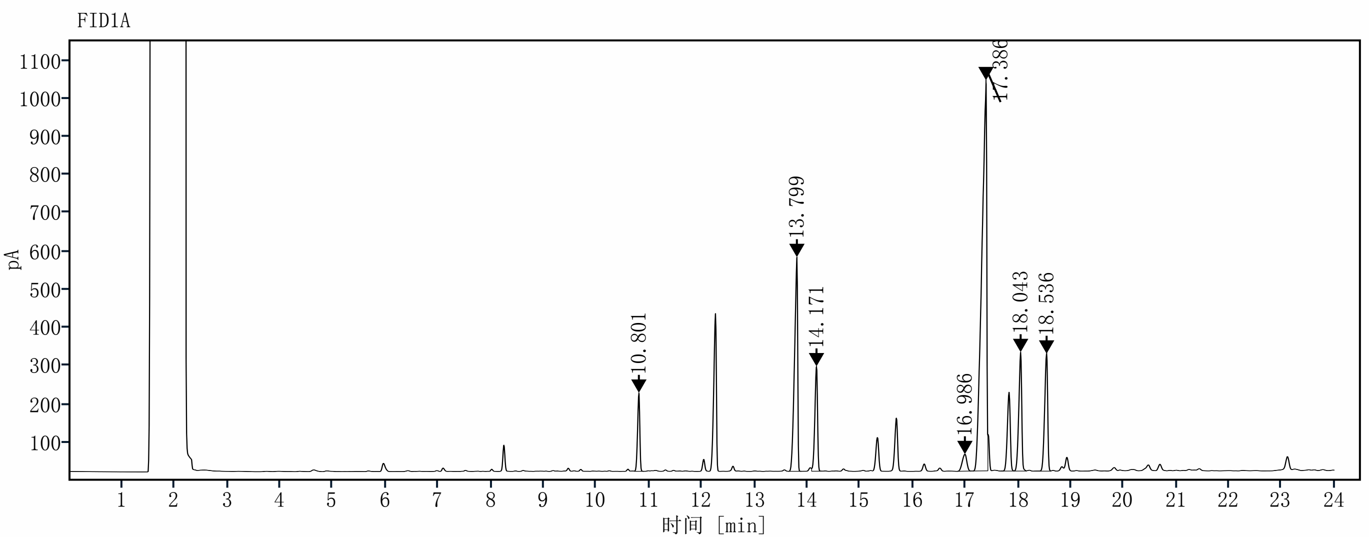

信号: FID1A

名称	化合物 浓度	化合物 含量	保留时间 [min]	类型	峰面积	峰面积%
			10.801	VB	600.54	4.31
			13.799	BB	2304.23	16.53
			14.171	VB	969.56	6.96
			16.986	BB	264.97	1.90
			17.386	VV	6570.57	47.15
			18.043	BV m	1996.96	14.33
			18.536	BV	1228.89	8.82
				总和	13935.72	

单次进样报告

数据文件:	6696.A Histidine20200610 125634.dx		
序列名称:	6696.A profile 1	项目名称:	脂肪酸
样品名称:	6696.A Histidine	操作者:	系统
仪器:	7890B	进样日期:	2020-06-10 13:01:11+08:00
进样体积:	1.000	位置:	108
采集方法:	脂肪酸测定方法25min.amx	类型:	样品
处理方法:	GC_LC<br>面积百分比_DefaultMethod.pmx	样品含量:	0.00
手动修改:	手动积分		

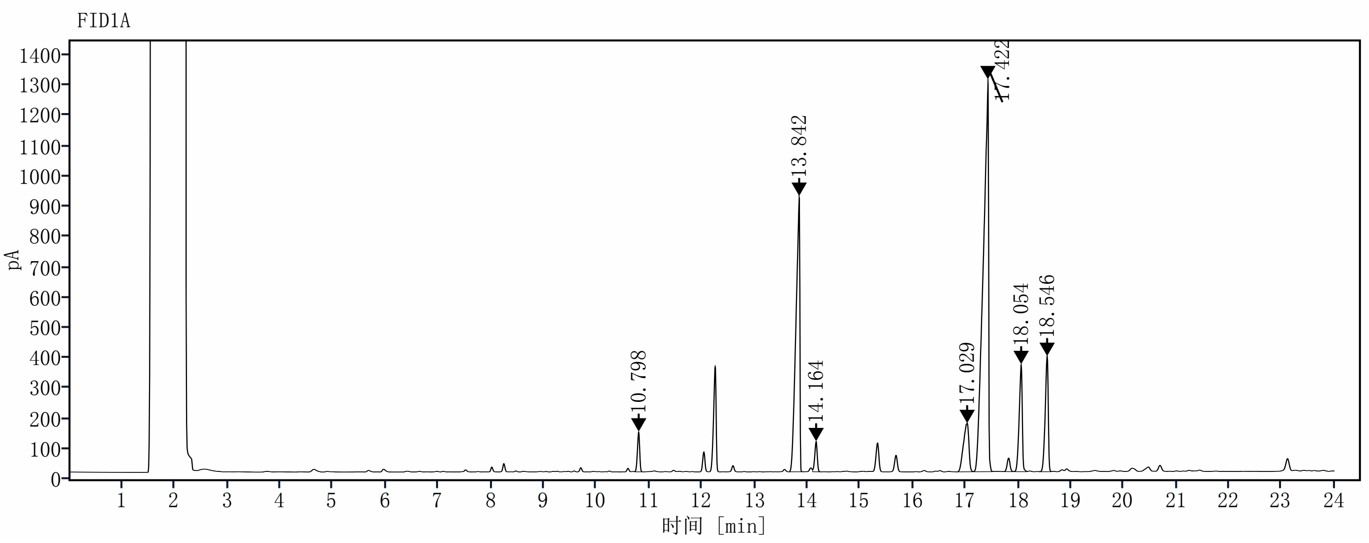

信号: FID1A

名称	化合物 浓度	化合物 含量	保留时间 [min]	类型	峰面积	峰面积%
			10.798	VB	388.51	2.09
			13.842	BB	4620.09	24.87
			14.164	VB	324.99	1.75
			17.029	BV	1147.57	6.18
			17.422	VV	8982.44	48.35
			18.054	BV m	1596.09	8.59
			18.546	VV	1518.20	8.17
				总和	18577.89	

数据文件:	6696.A Ph. alanine20200610 132513.dx		
序列名称:	6696.A profile 1	项目名称:	脂肪酸
样品名称:	6696.A Ph. alanine	操作者:	系统
仪器:	7890B	进样日期:	2020-06-10 13:29:36+08:00
进样体积:	1.000	位置:	109
采集方法:	脂肪酸测定方法25min.amx	类型:	样品
处理方法:	GC_LC<br>面积百分比_DefaultMethod.pmx	样品含量:	0.00
手动修改:	手动积分		

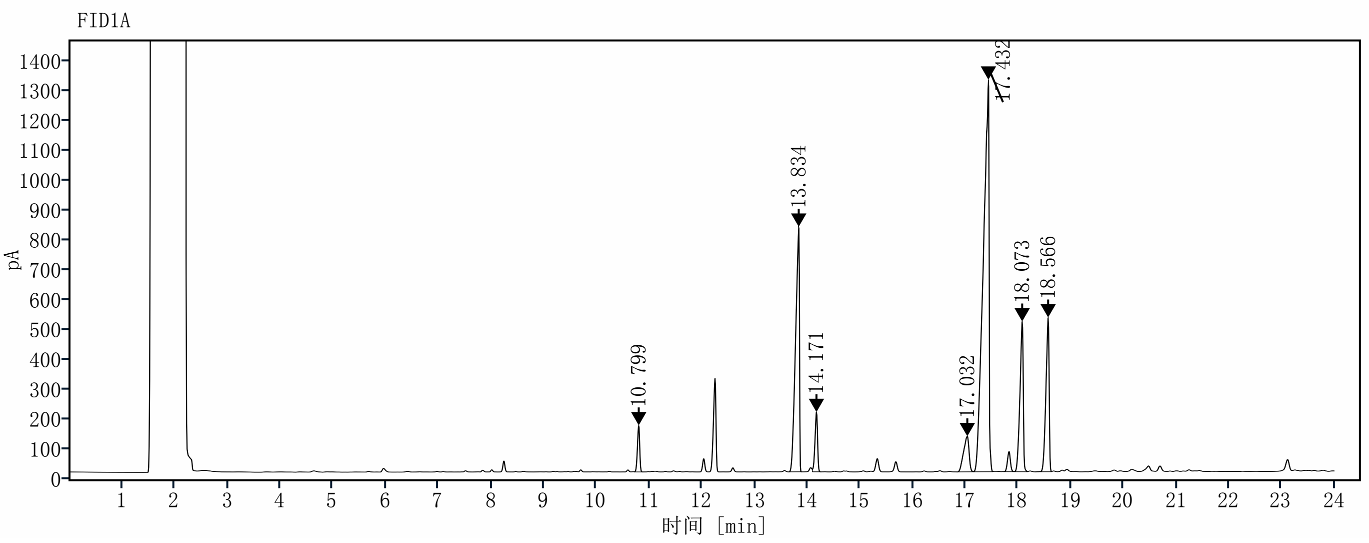

信号: FID1A

名称	化合物 浓度	化合物 含量	保留时间 [min]	类型	峰面积	峰面积%
			10.799	VB	460.99	2.23
			13.834	BB	4138.67	20.02
			14.171	VB	670.58	3.24
			17.032	BV	855.88	4.14
			17.432	VV	9930.92	48.03
			18.073	VV m	2448.26	11.84
			18.566	BV	2170.51	10.50
				总和	20675.82	

单次进样报告

Agilent Technologies

数据文件:	6696.A Cystine20200610 135338.dx		
序列名称:	6696.A profile 1	项目名称:	脂肪酸
样品名称:	6696.A Cystine	操作者:	系统
仪器:	7890B	进样日期:	2020-06-10 13:58:06+08:00
进样体积:	1.000	位置:	110
采集方法:	脂肪酸测定方法25min.amx	类型:	样品
处理方法:	GC_LC<br>面积百分比_DefaultMethod.pmx	样品含量:	0.00
手动修改:	手动积分		

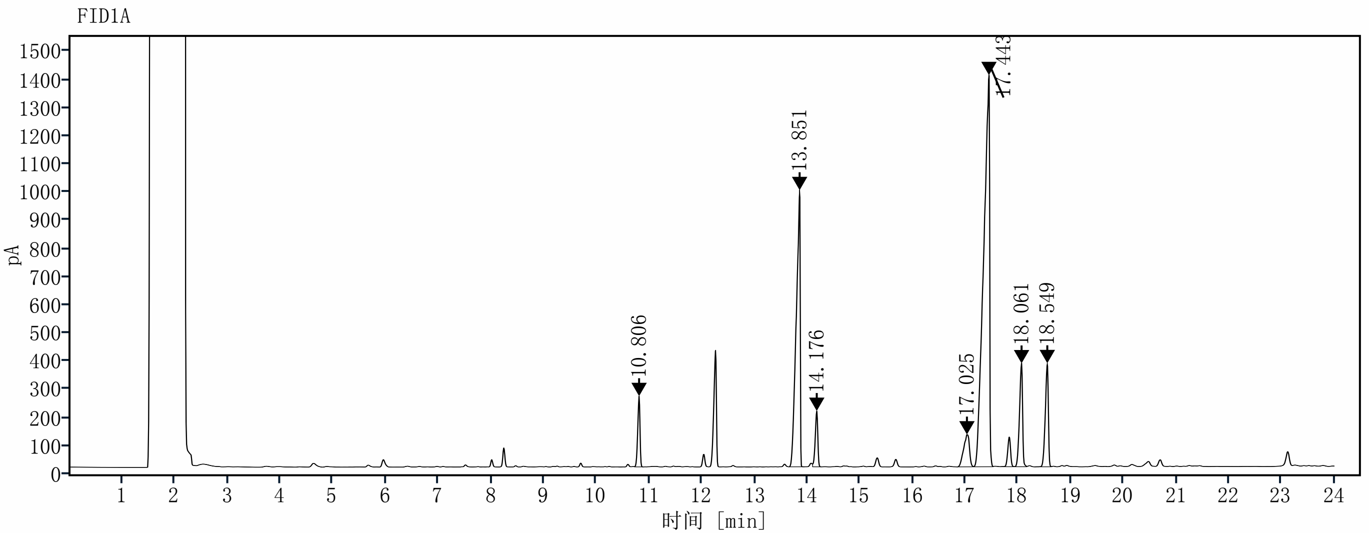

信号: FID1A

名称	化合物 浓度	化合物 含量	保留时间 [min]	类型	峰面积	峰面积%
			10.806	VB	741.45	3.44
			13.851	BB	5091.19	23.60
			14.176	VB	662.61	3.07
			17.025	BV	871.86	4.04
			17.443	VV	10872.96	50.41
			18.061	BV m	1873.93	8.69
			18.549	BV	1456.26	6.75
				总和	21570.25	

单次进样报告

数据文件:	6696.A Glutamic acid20200610 142208.dx		
序列名称:	6696.A profile 1	项目名称:	脂肪酸
样品名称:	6696.A Glutamic acid	操作者:	系统
仪器:	7890B	进样日期:	2020-06-10 14:26:33+08:00
进样体积:	1.000	位置:	111
采集方法:	脂肪酸测定方法25min.amx	类型:	样品
处理方法:	GC_LC<br>面积百分比_DefaultMethod.pmx	样品含量:	0.00
手动修改:	手动积分		

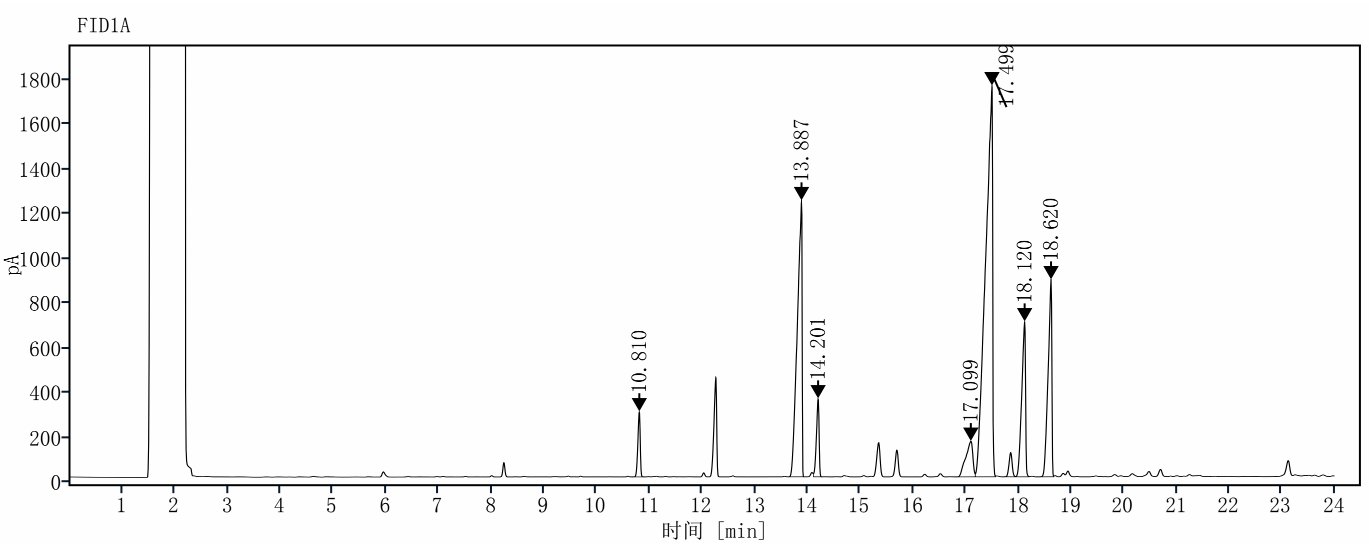

信号: FID1A

名称	化合物 浓度	化合物 含量	保留时间 [min]	类型	峰面积	峰面积%
			10.810	VB	902.97	2.53
			13.887	VB	7717.80	21.62
			14.201	VB	1217.41	3.41
			17.099	BV	1477.82	4.14
			17.499	VV	16186.60	45.35
			18.120	BV m	3885.07	10.89
			18.620	BV	4303.99	12.06
				总和	35691.65	

单次进样报告

数据文件:	6696.A Methionine20200610 145035.dx		
序列名称:	6696.A profile 1	项目名称:	脂肪酸
样品名称:	6696.A Methionine	操作者:	系统
仪器:	7890B	进样日期:	2020-06-10 14:55:02+08:00
进样体积:	1.000	位置:	112
采集方法:	脂肪酸测定方法25min.amx	类型:	样品
处理方法:	GC_LC<br>面积百分比_DefaultMethod.pmx	样品含量:	0.00
手动修改:	手动积分		

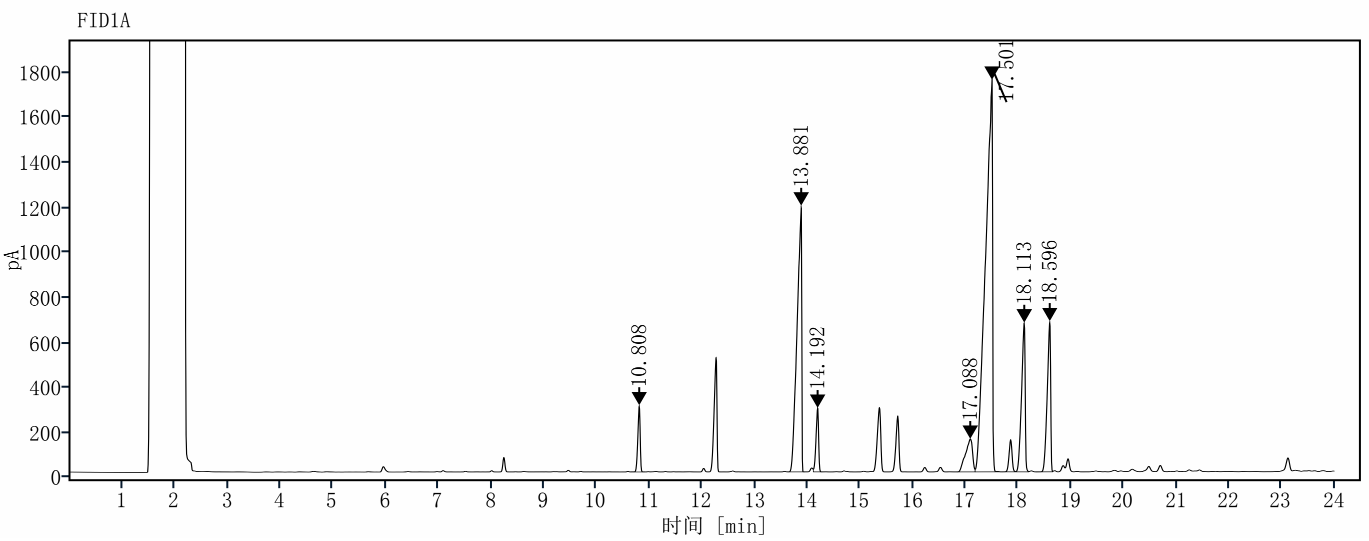

信号: FID1A

名称	化合物 浓度	化合物 含量	保留时间 [min]	类型	峰面积	峰面积%
			10.808	VB	900.48	2.72
			13.881	VB	7145.35	21.58
			14.192	VB	950.14	2.87
			17.088	BV	1352.69	4.09
			17.501	VB	16099.94	48.62
			18.113	BV m	3691.45	11.15
			18.596	BV	2972.19	8.98
				总和	33112.24	

单次进样报告

**数据文件:**	6696.A Glycine20200610 151903.dx		
**序列名称:**	6696.A profile 1	**项目名称:**	脂肪酸
**样品名称:**	6696.A Glycine	**操作者:**	系统
**仪器:**	7890B	**进样日期:**	2020-06-10 15:23:29+08:00
**进样体积:**	1.000	**位置:**	113
**采集方法:**	脂肪酸测定方法25min.amx	**类型:**	样品
**处理方法:**	GC_LC<br>面积百分比_DefaultMethod.pmx	**样品含量:**	0.00
**手动修改:**	手动积分		

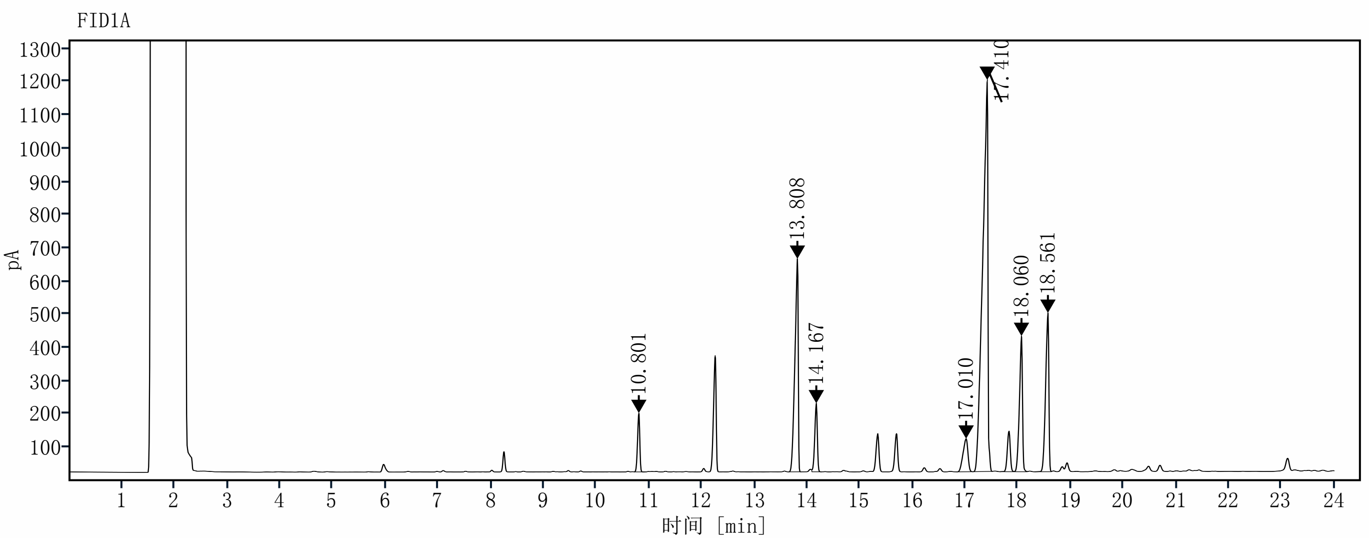

信号: FID1A

名称	化合物 浓度	化合物 含量	保留时间 [min]	类型	峰面积	峰面积%
			10.801	VB	520.62	3.09
			13.808	BB	2783.20	16.54
			14.167	VB	693.69	4.12
			17.010	BB	639.16	3.80
			17.410	BB	8062.44	47.92
			18.060	BV m	2109.20	12.54
			18.561	BV	2018.03	11.99
				总和	16826.33	

数据文件:	6696.A Leucine20200610 154731.dx		
序列名称:	6696.A profile 1	项目名称:	脂肪酸
样品名称:	6696.A Leucine	操作者:	系统
仪器:	7890B	进样日期:	2020-06-10 15:51:53+08:00
进样体积:	1.000	位置:	114
采集方法:	脂肪酸测定方法25min.amx	类型:	样品
处理方法:	GC_LC<br>面积百分比_DefaultMethod.pmx	样品含量:	0.00
手动修改:	手动积分		

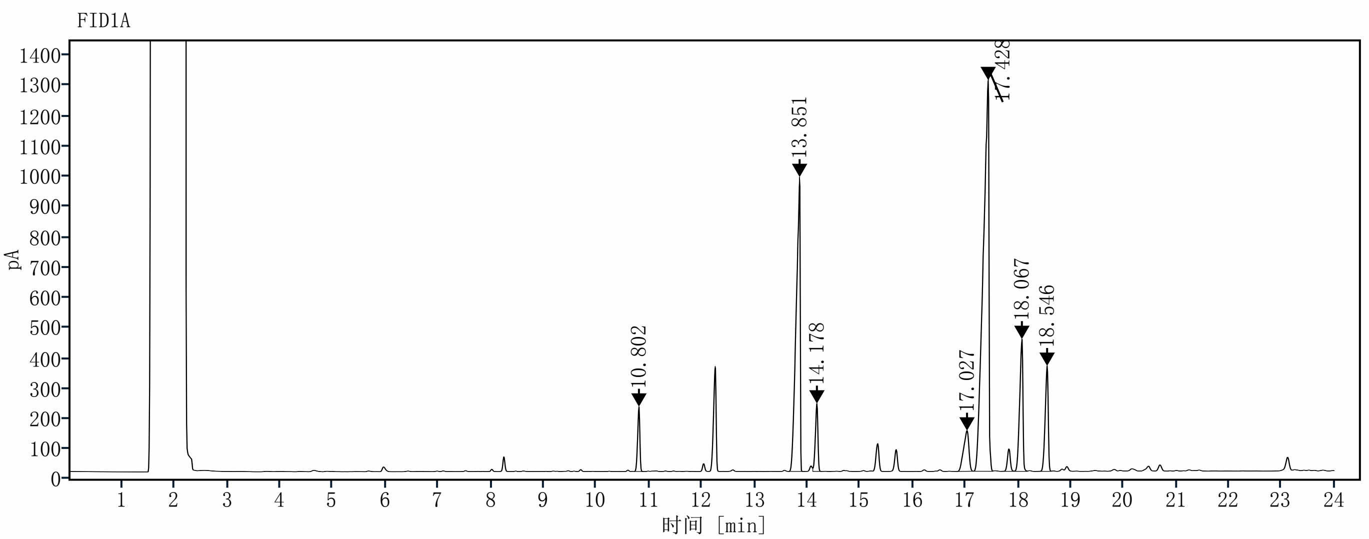

信号: FID1A

名称	化合物 浓度	化合物 含量	保留时间 [min]	类型	峰面积	峰面积%
			10.802	VB	623.17	3.02
			13.851	BB	5099.12	24.71
			14.178	VB	747.81	3.62
			17.027	BB	934.63	4.53
			17.428	BV	9697.55	47.00
			18.067	BV m	2145.15	10.40
			18.546	BV	1384.46	6.71
				总和	20631.89	

单次进样报告

数据文件:	6696.A Lycine20200610 161554.dx		
序列名称:	6696.A profile 1	项目名称:	脂肪酸
样品名称:	6696.A Lycine	操作者:	系统
仪器:	7890B	进样日期:	2020-06-10 16:20:17+08:00
进样体积:	1.000	位置:	115
采集方法:	脂肪酸测定方法25min.amx	类型:	样品
处理方法:	GC_LC<br>面积百分比_DefaultMethod.pmx	样品含量:	0.00
手动修改:	手动积分		

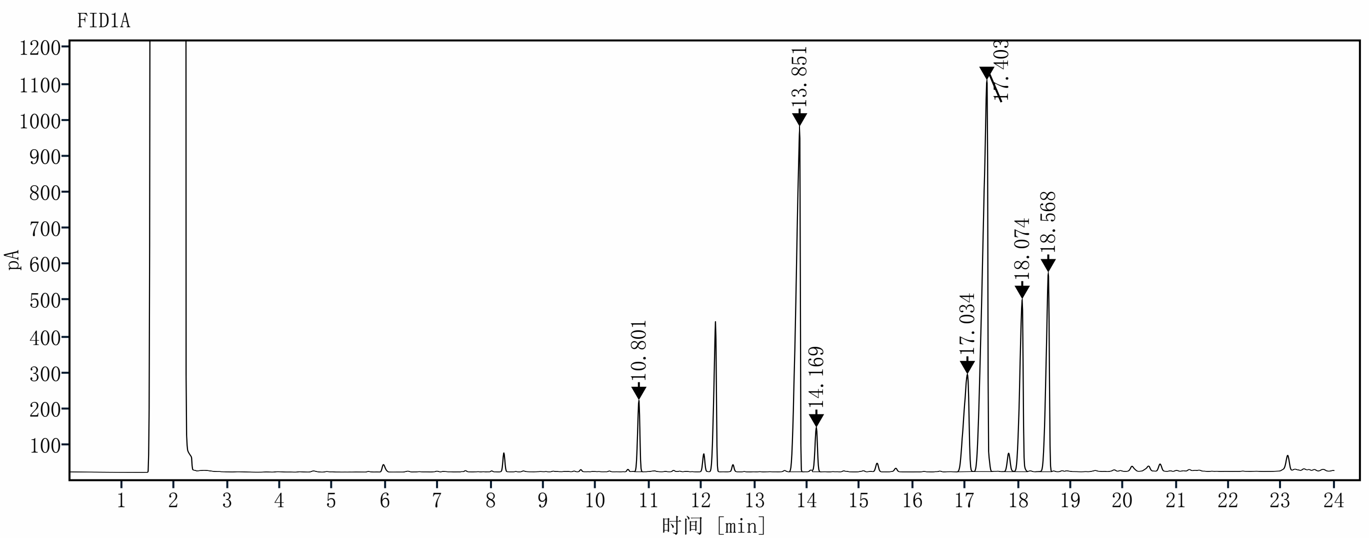

信号: FID1A

名称	化合物 浓度	化合物 含量	保留时间 [min]	类型	峰面积	峰面积%
			10.801	VB	597.59	3.00
			13.851	BV	5122.26	25.68
			14.169	VB	406.01	2.04
			17.034	BB	1838.20	9.22
			17.403	BV	7299.12	36.60
			18.074	BV m	2323.41	11.65
			18.568	BV	2357.33	11.82
				总和	19943.92	

单次进样报告

数据文件:	6696.A Iso-leucine20200610 164418.dx		
序列名称:	6696.A profile 1	项目名称:	脂肪酸
样品名称:	6696.A Iso-leucine	操作者:	系统
仪器:	7890B	进样日期:	2020-06-10 16:48:42+08:00
进样体积:	1.000	位置:	116
采集方法:	脂肪酸测定方法25min.amx	类型:	样品
处理方法:	GC_LC<br>面积百分比_DefaultMethod.pmx	样品含量:	0.00
手动修改:	手动积分		

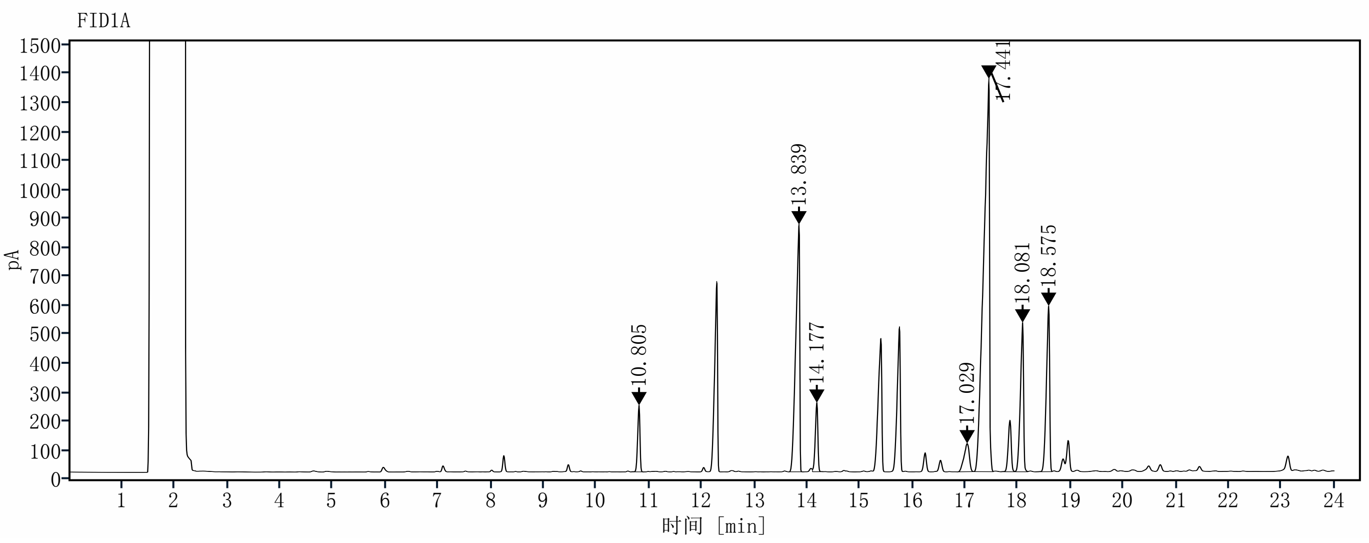

信号: FID1A

名称	化合物 浓度	化合物 含量	保留时间 [min]	类型	峰面积	峰面积%
			10.805	VB	696.09	3.14
			13.839	BB	4335.59	19.57
			14.177	VB	806.72	3.64
			17.029	BB	678.15	3.06
			17.441	BV	10334.34	46.64
			18.081	BV m	2851.07	12.87
			18.575	BV	2453.55	11.07
				总和	22155.50	

单次进样报告

Agilent Technologies

**数据文件:**	6696.A Aspargine20200611 104537.dx		
**序列名称:**	6696.A profile 2	**项目名称:**	脂肪酸
**样品名称:**	6696.A Aspargine	**操作者:**	系统
**仪器:**	7890B	**进样日期:**	2020-06-11 10:50:08+08:00
**进样体积:**	1.000	**位置:**	102
**采集方法:**	脂肪酸测定方法25min.amx	**类型:**	样品
**处理方法:**	GC_LC<br>面积百分比_DefaultMethod.pmx	**样品含量:**	0.00
**手动修改:**	手动积分		

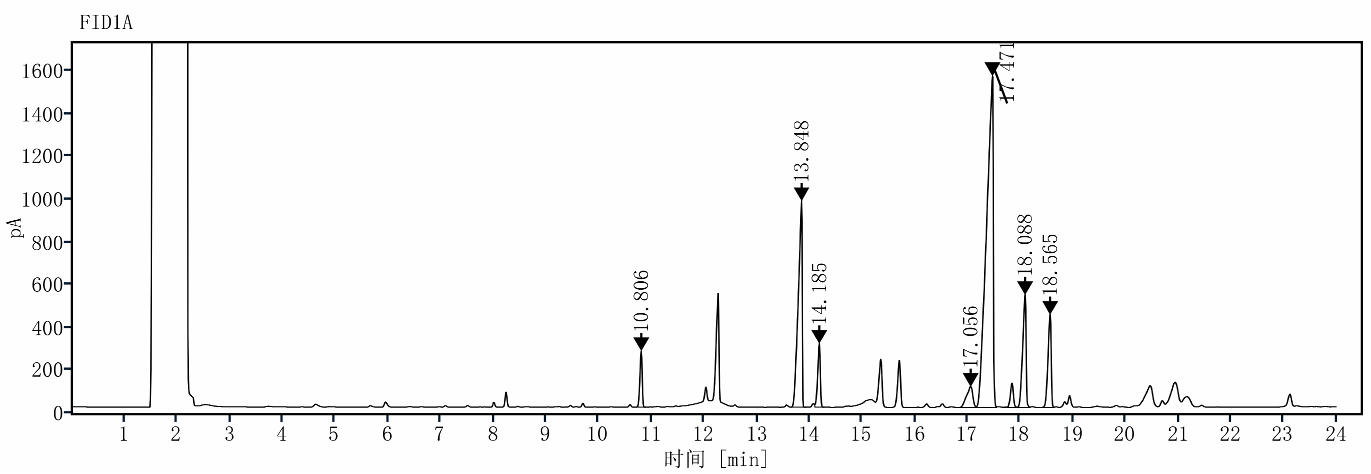

信号: FID1A

名称	化合物浓度	化合物含量	保留时间 [min]	类型	峰宽 [min]	高度	峰面积	峰面积%
			10.806	VB	0.17	263.84	784.89	3.06
			13.848	BB	0.29	964.74	4957.32	19.33
			14.185	VB	0.18	299.01	1030.93	4.02
			17.056	BV	0.32	97.36	786.38	3.07
			17.471	VV	0.38	1550.50	13535.97	52.77
			18.088	BV m	0.06	527.18	2767.32	10.79
			18.565	VV	0.25	434.46	1786.13	6.96
						总和	25648.95	

单次进样报告

Agilent Technologies

数据文件:	6696.A Proline20200611 111410.dx		
序列名称:	6696.A profile 2	项目名称:	脂肪酸
样品名称:	6696.A Proline	操作者:	系统
仪器:	7890B	进样日期:	2020-06-11 11:18:37+08:00
进样体积:	1.000	位置:	103
采集方法:	脂肪酸测定方法25min.amx	类型:	样品
处理方法:	GC_LC<br>面积百分比_DefaultMethod.pmx	样品含量:	0.00
手动修改:	手动积分		

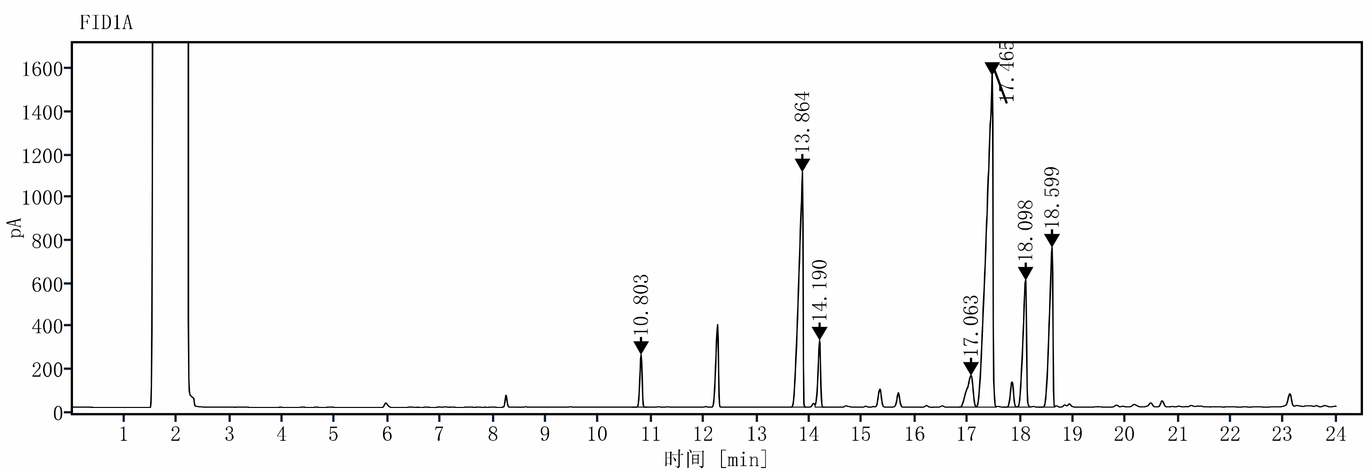

信号: FID1A

名称	化合物浓度	化合物含量	保留时间 [min]	类型	峰宽 [min]	高度	峰面积	峰面积%
			10.803	VB	0.28	245.24	745.70	2.59
			13.864	VV	0.29	1095.67	6133.75	21.27
			14.190	VB	0.18	311.72	1056.45	3.66
			17.063	BV	0.34	147.65	1209.13	4.19
			17.465	VV	0.38	1544.75	12889.09	44.69
			18.098	BV m	0.07	590.64	3250.65	11.27
			18.599	BV	0.32	745.03	3556.22	12.33
						总和	28841.00	

单次进样报告

Agilent Technologies

**数据文件:**	6696.A Control20200611 114238.dx		
**序列名称:**	6696.A profile 2	**项目名称:**	脂肪酸
**样品名称:**	6696.A Control	**操作者:**	系统
**仪器:**	7890B	**进样日期:**	2020-06-11 11:47:05+08:00
**进样体积:**	1.000	**位置:**	104
**采集方法:**	脂肪酸测定方法25min.amx	**类型:**	样品
**处理方法:**	GC_LC<br>面积百分比_DefaultMethod.pmx	**样品含量:**	0.00
**手动修改:**	手动积分		

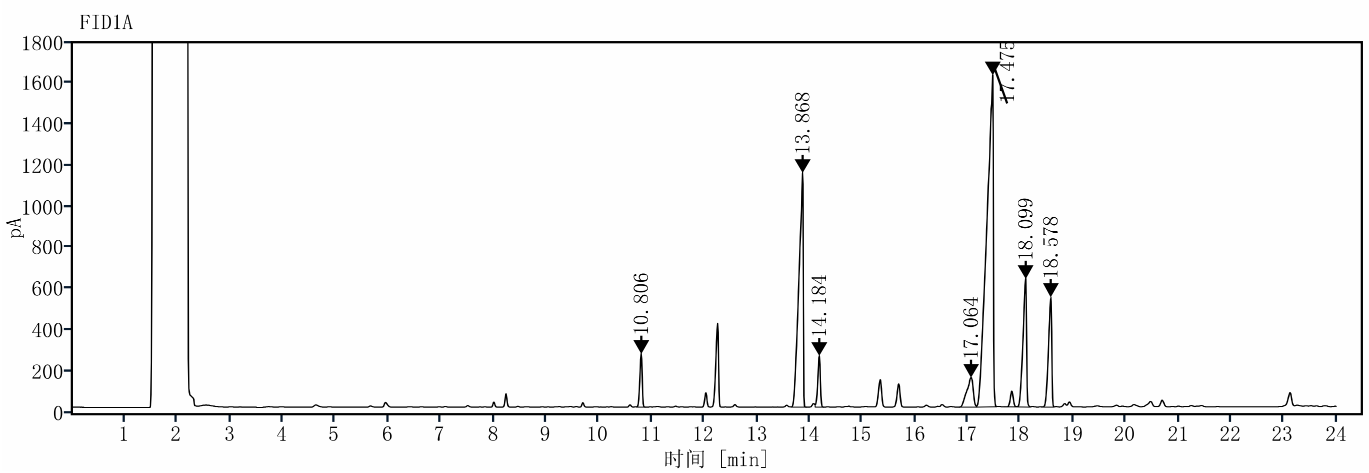

信号: FID1A

名称	化合物浓度	化合物含量	保留时间 [min]	类型	峰宽 [min]	高度	峰面积	峰面积%
			10.806	VB	0.17	260.61	796.45	2.78
			13.868	VB	0.30	1140.70	6551.91	22.90
			14.184	VB	0.16	250.20	843.45	2.95
			17.064	BV	0.33	144.20	1196.88	4.18
			17.475	VV	0.38	1615.82	13765.71	48.12
			18.099	BV m	0.07	624.63	3178.37	11.11
			18.578	VV	0.25	536.23	2272.15	7.94
						总和	28604.92	

单次进样报告

Agilent Technologies

数据文件:	11616.A Serine20200611 121108.dx		
序列名称:	6696.A profile 2	项目名称:	脂肪酸
样品名称:	11616.A Serine	操作者:	系统
仪器:	7890B	进样日期:	2020-06-11 12:15:33+08:00
进样体积:	1.000	位置:	105
采集方法:	脂肪酸测定方法25min.amx	类型:	样品
处理方法:	GC_LC<br>面积百分比_DefaultMethod.pmx	样品含量:	0.00
手动修改:	手动积分		

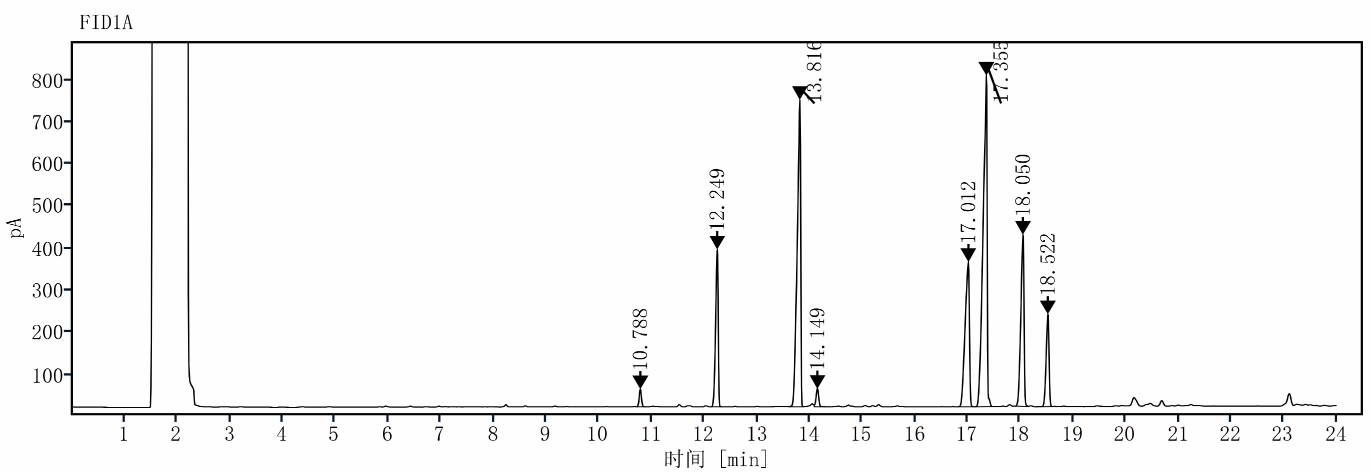

信号: FID1A

名称	化合物浓度	化合物含量	保留时间 [min]	类型	峰宽 [min]	高度	峰面积	峰面积%
			10.788	VB	0.19	42.64	123.74	0.92
			12.249	VV	0.21	373.76	1253.32	9.30
			13.816	VV	0.30	727.96	3326.32	24.68
			14.149	VB	0.30	43.45	148.47	1.10
			17.012	BB	0.31	343.32	1915.36	14.21
			17.355	BV	0.34	785.07	4181.80	31.03
			18.050	VV	0.25	407.17	1705.06	12.65
			18.522	BV	0.32	219.66	822.63	6.10
						总和	13476.70	

单次进样报告

Agilent Technologies

**数据文件:**	11616.A Alanine20200611 123935.dx		
**序列名称:**	6696.A profile 2	**项目名称:**	脂肪酸
**样品名称:**	11616.A Alanine	**操作者:**	系统
**仪器:**	7890B	**进样日期:**	2020-06-11 12:44:06+08:00
**进样体积:**	1.000	**位置:**	106
**采集方法:**	脂肪酸测定方法25min.amx	**类型:**	样品
**处理方法:**	GC_LC 面积百分比_DefaultMethod.pmx	**样品含量:**	0.00
**手动修改:**	手动积分		

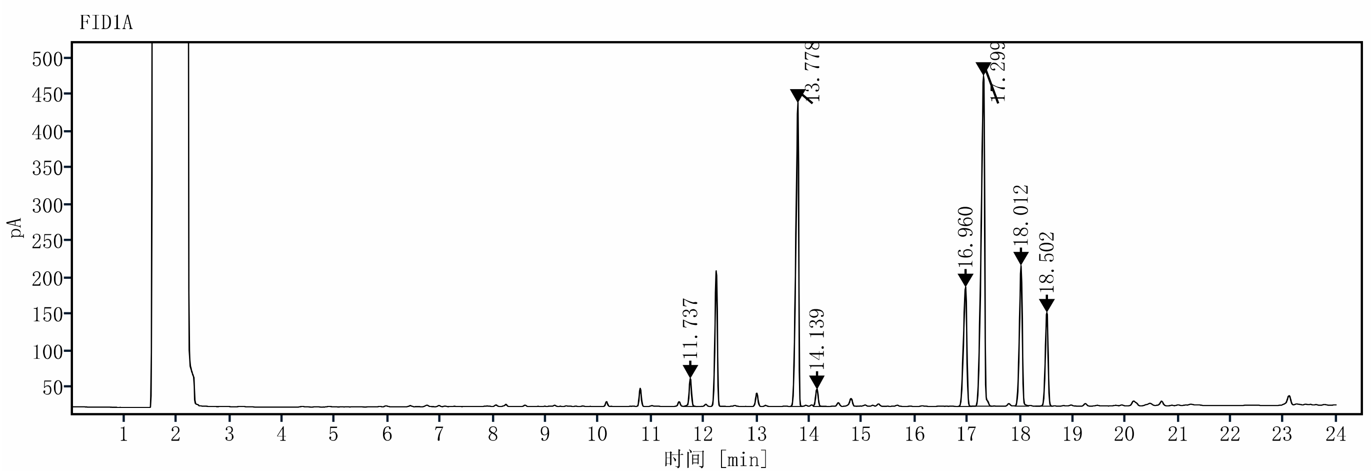

信号: FID1A

名称	化合物浓度	化合物含量	保留时间 [min]	类型	峰宽 [min]	高度	峰面积	峰面积%
			11.737	VB	0.26	38.41	122.21	2.12
			13.778	BB	0.25	414.96	1570.10	27.29
			14.139	VB	0.30	23.67	81.18	1.41
			16.960	BB	0.26	163.10	728.60	12.66
			17.299	BV	0.38	451.94	2053.99	35.70
			18.012	VV	0.26	192.54	732.42	12.73
			18.502	BV	0.32	128.24	465.05	8.08
						总和	5753.55	

数据文件:	11616.A Arginine20200611 130809.dx		
序列名称:	6696.A profile 2	项目名称:	脂肪酸
样品名称:	11616.A Arginine	操作者:	系统
仪器:	7890B	进样日期:	2020-06-11 13:12:36+08:00
进样体积:	1.000	位置:	107
采集方法:	脂肪酸测定方法25min.amx	类型:	样品
处理方法:	GC_LC<br>面积百分比_DefaultMethod.pmx	样品含量:	0.00
手动修改:	手动积分		

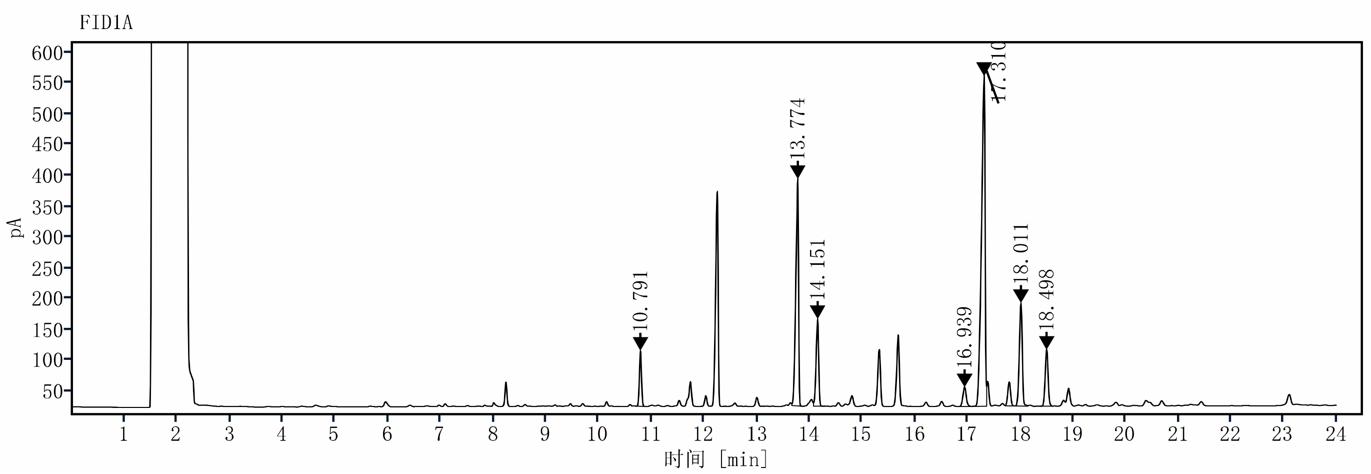

信号: FID1A

名称	化合物浓度	化合物含量	保留时间 [min]	类型	峰宽 [min]	高度	峰面积	峰面积%
			10.791	BB	0.18	90.43	254.07	4.33
			13.774	VB	0.18	368.63	1325.87	22.58
			14.151	VB	0.22	141.64	476.21	8.11
			16.939	BB	0.24	31.48	139.27	2.37
			17.310	BV	0.28	535.63	2532.28	43.12
			18.011	VV m	0.06	167.44	794.93	13.54
			18.498	VV	0.25	91.87	349.62	5.95
						总和	5872.25	

单次进样报告

Agilent Technologies

**数据文件:**	11616.A Glutamine20200611 133638.dx		
**序列名称:**	6696.A profile 2	**项目名称:**	脂肪酸
**样品名称:**	11616.A Glutamine	**操作者:**	系统
**仪器:**	7890B	**进样日期:**	2020-06-11 13:41:05+08:00
**进样体积:**	1.000	**位置:**	108
**采集方法:**	脂肪酸测定方法25min.amx	**类型:**	样品
**处理方法:**	GC_LC 面积百分比_DefaultMethod.pmx	**样品含量:**	0.00
**手动修改:**	手动积分		

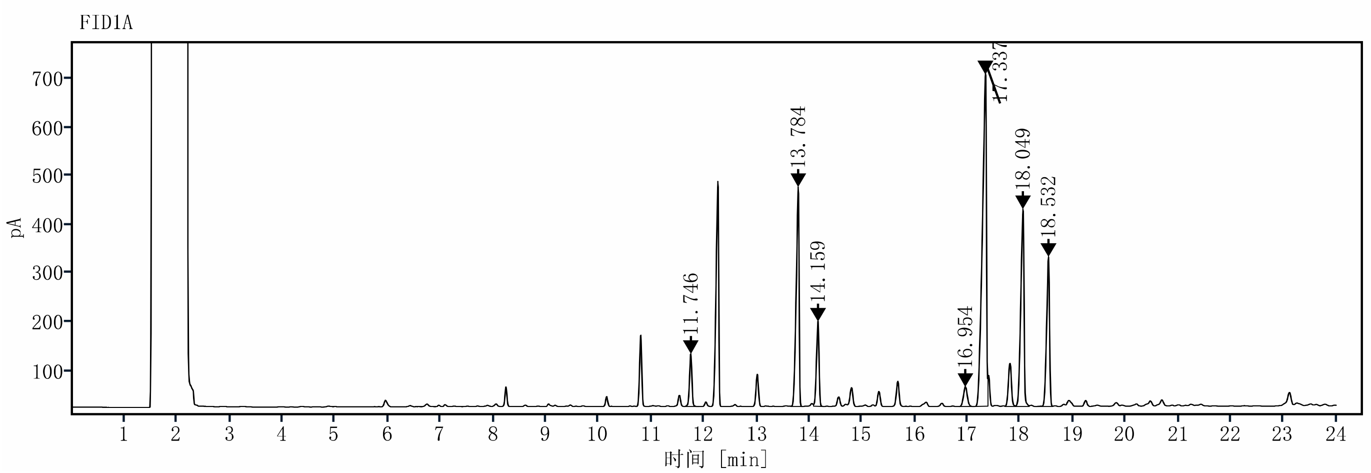

信号: FID1A

名称	化合物浓度	化合物含量	保留时间 [min]	类型	峰宽 [min]	高度	峰面积	峰面积%
			11.746	VB	0.28	108.75	348.85	3.54
			13.784	VB	0.22	451.85	1741.35	17.68
			14.159	VB	0.19	174.64	592.02	6.01
			16.954	BB	0.26	40.80	206.69	2.10
			17.337	BV	0.29	683.22	3692.60	37.48
			18.049	VV m	0.06	406.41	2085.70	21.17
			18.532	BV	0.33	308.18	1184.80	12.03
						总和	9852.01	

**数据文件:**	11616.A Tryptophane20200611 140507.dx		
**序列名称:**	6696.A profile 2	**项目名称:**	脂肪酸
**样品名称:**	11616.A Tryptophane	**操作者:**	系统
**仪器:**	7890B	**进样日期:**	2020-06-11 14:09:41+08:00
**进样体积:**	1.000	**位置:**	109
**采集方法:**	脂肪酸测定方法25min.amx	**类型:**	样品
**处理方法:**	GC_LC 面积百分比_DefaultMethod.pmx	**样品含量:**	0.00
**手动修改:**	手动积分		

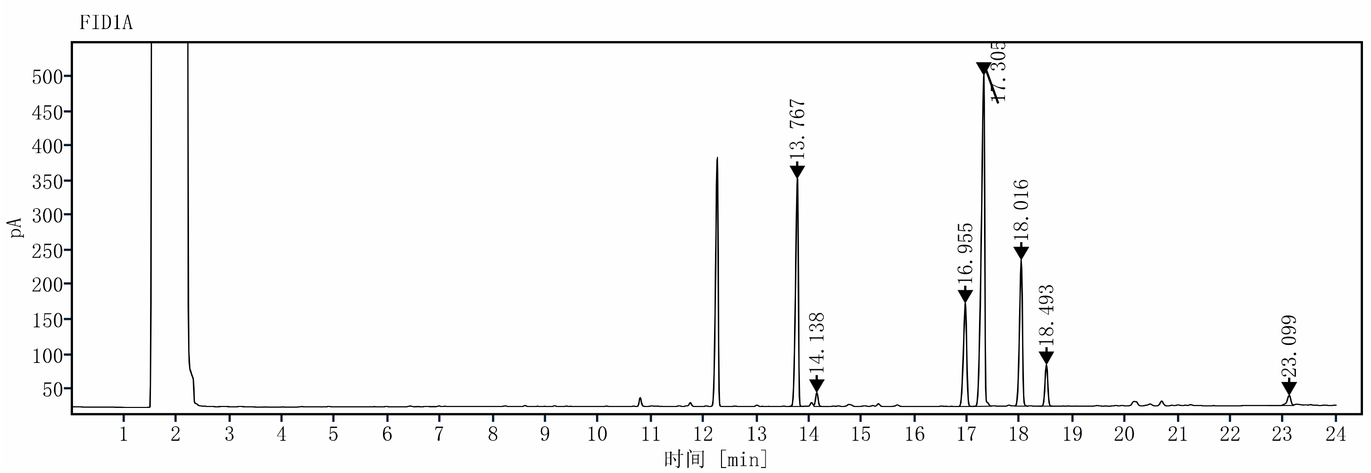

信号: FID1A

名称	化合物浓度	化合物含量	保留时间 [min]	类型	峰宽 [min]	高度	峰面积	峰面积%
			13.767	BB	0.29	326.90	1211.95	23.60
			14.138	VB	0.28	19.51	66.98	1.30
			16.955	BB	0.26	147.99	645.65	12.57
			17.305	BV	0.38	475.78	2099.24	40.88
			18.016	VV	0.24	210.28	814.36	15.86
			18.493	VV	0.25	59.28	219.76	4.28
			23.099	BV	0.49	14.52	77.51	1.51
						总和	5135.46	

单次进样报告

Agilent Technologies

**数据文件:**	11616.A Tyrosine20200611 143343.dx		
**序列名称:**	6696.A profile 2	**项目名称:**	脂肪酸
**样品名称:**	11616.A Tyrosine	**操作者:**	系统
**仪器:**	7890B	**进样日期:**	2020-06-11 14:38:12+08:00
**进样体积:**	1.000	**位置:**	110
**采集方法:**	脂肪酸测定方法25min.amx	**类型:**	样品
**处理方法:**	GC_LC<br>面积百分比_DefaultMethod.pmx	**样品含量:**	0.00
**手动修改:**	手动积分		

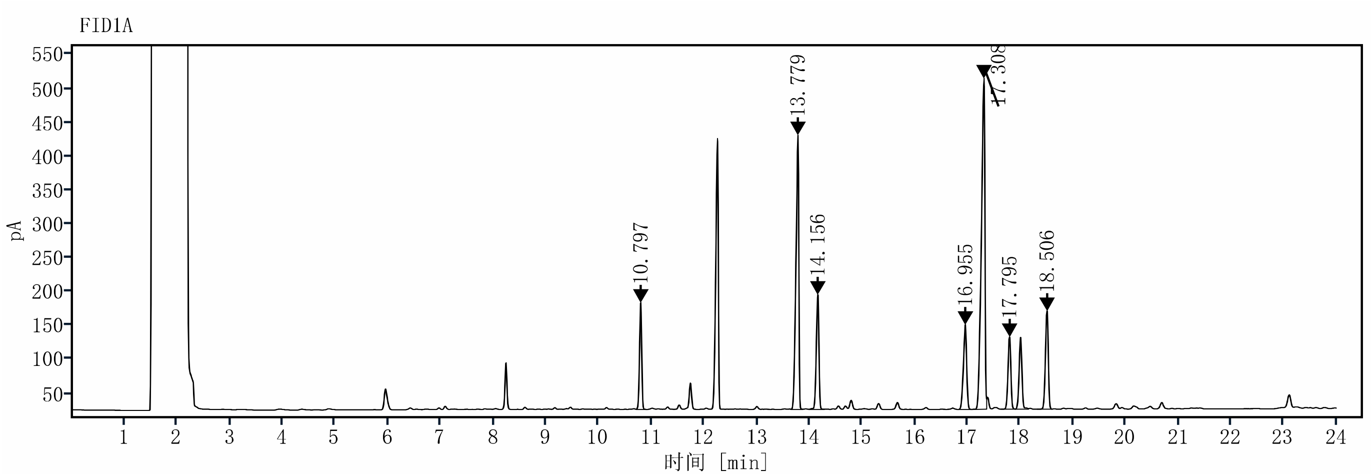

信号: FID1A

名称	化合物浓度	化合物含量	保留时间 [min]	类型	峰宽 [min]	高度	峰面积	峰面积%
			10.797	VB	0.20	157.68	456.73	6.83
			13.779	VV	0.23	405.21	1523.30	22.77
			14.156	BV	0.29	169.42	587.51	8.78
			16.955	BB	0.25	125.22	532.42	7.96
			17.308	BV	0.28	488.34	2245.46	33.56
			17.795	BV m	0.09	107.07	798.00	11.93
			18.506	VV	0.28	145.36	547.34	8.18
						总和	6690.76	

单次进样报告

Agilent Technologies

**数据文件:**	11616.A Valine20200611 150213.dx		
**序列名称:**	6696.A profile 2	**项目名称:**	脂肪酸
**样品名称:**	11616.A Valine	**操作者:**	系统
**仪器:**	7890B	**进样日期:**	2020-06-11 15:06:41+08:00
**进样体积:**	1.000	**位置:**	111
**采集方法:**	脂肪酸测定方法25min.amx	**类型:**	样品
**处理方法:**	GC_LC 面积百分比_DefaultMethod.pmx	**样品含量:**	0.00
**手动修改:**	手动积分		

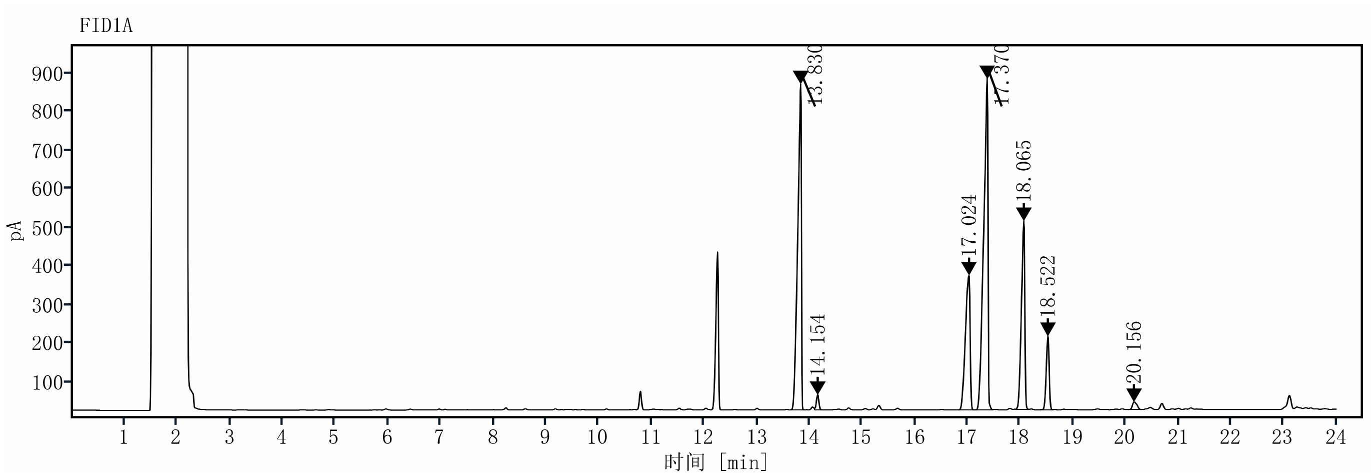

信号: FID1A

名称	化合物浓度	化合物含量	保留时间 [min]	类型	峰宽 [min]	高度	峰面积	峰面积%
			13.830	BV	0.27	845.06	4045.30	28.47
			14.154	VB	0.13	39.02	125.31	0.88
			17.024	BB	0.32	349.11	2151.67	15.14
			17.370	BV	0.35	858.57	4861.31	34.22
			18.065	VV	0.25	489.73	2172.32	15.29
			18.522	BV	0.32	191.28	732.57	5.16
			20.156	BV	0.27	19.99	118.68	0.84
						总和	14207.17	

单次进样报告

Agilent Technologies

**数据文件:**	11616.A Histidine20200611 153043.dx		
**序列名称:**	6696.A profile 2	**项目名称:**	脂肪酸
**样品名称:**	11616.A Histidine	**操作者:**	系统
**仪器:**	7890B	**进样日期:**	2020-06-11 15:35:14+08:00
**进样体积:**	1.000	**位置:**	112
**采集方法:**	脂肪酸测定方法25min.amx	**类型:**	样品
**处理方法:**	GC_LC<br>面积百分比_DefaultMethod.pmx	**样品含量:**	0.00
**手动修改:**	手动积分		

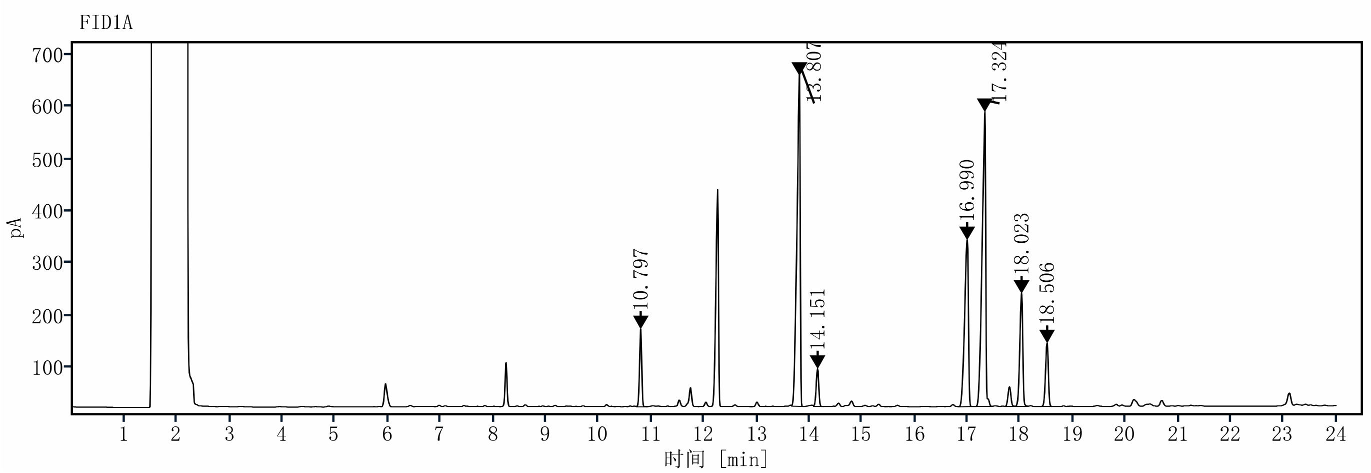

信号: FID1A

名称	化合物浓度	化合物含量	保留时间 [min]	类型	峰宽 [min]	高度	峰面积	峰面积%
			10.797	VB	0.19	148.47	427.84	4.70
			13.807	VB	0.23	632.00	2748.74	30.21
			14.151	VB	0.20	72.34	242.42	2.66
			16.990	BB	0.26	318.09	1578.41	17.35
			17.324	BV	0.37	562.51	2666.54	29.31
			18.023	BV m	0.06	215.76	982.01	10.79
			18.506	BV	0.33	121.23	451.91	4.97
						总和	9097.88	

单次进样报告

Agilent Technologies

数据文件:	11616.A Ph. alanine20200612 084536.dx		
序列名称:	6696.A profile 3	项目名称:	脂肪酸
样品名称:	11616.A Ph. alanine	操作者:	系统
仪器:	7890B	进样日期:	2020-06-12 10:32:49+08:00
进样体积:	1.000	位置:	101
采集方法:	脂肪酸测定方法25min.amx	类型:	样品
处理方法:	GC_LC<br>面积百分比_DefaultMethod.pmx	样品含量:	0.00
手动修改:	手动积分		

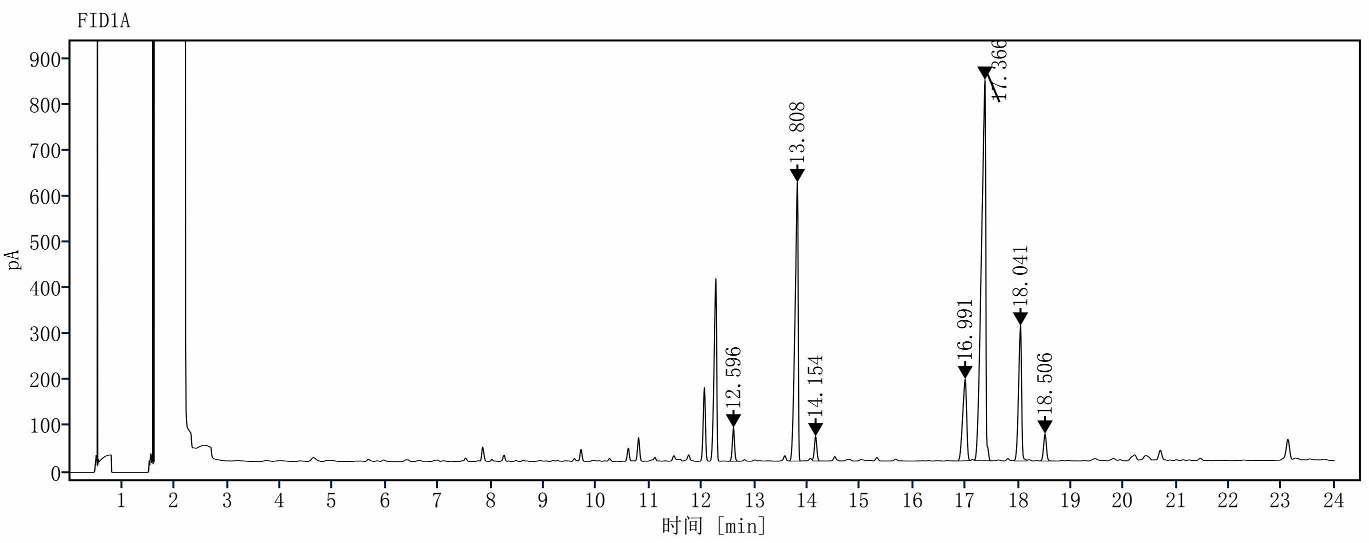

信号: FID1A

名称	化合物 浓度	化合物 含量	保留时间 [min]	类型	峰面积	峰面积%
			12.596	BV	209.00	2.10
			13.808	VB	2499.89	25.07
			14.154	VB	174.57	1.75
			16.991	BB	927.09	9.30
			17.366	VB	4763.89	47.78
			18.041	VV	1172.11	11.76
			18.506	VV	224.47	2.25
				总和	9971.02	

单次进样报告

Agilent Technologies

**数据文件:**	11616.A Cystine20200612 105650.dx		
**序列名称:**	6696.A profile 3	**项目名称:**	脂肪酸
**样品名称:**	11616.A Cystine	**操作者:**	系统
**仪器:**	7890B	**进样日期:**	2020-06-12 11:01:18+08:00
**进样体积:**	1.000	**位置:**	102
**采集方法:**	脂肪酸测定方法25min.amx	**类型:**	样品
**处理方法:**	GC_LC<br>面积百分比_DefaultMethod.pmx	**样品含量:**	0.00
**手动修改:**	手动积分		

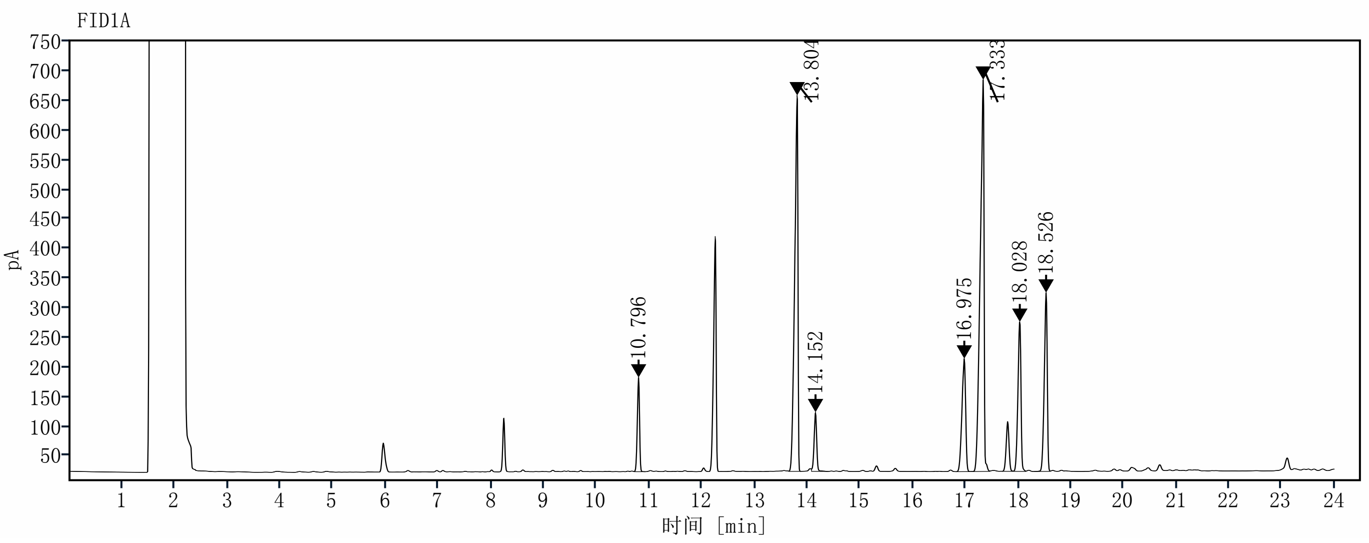

信号: FID1A

名称	化合物 浓度	化合物 含量	保留时间 [min]	类型	峰面积	峰面积%
			10.796	VB	465.98	4.53
			13.804	VB	2702.12	26.26
			14.152	VB	339.14	3.30
			16.975	BB	926.01	9.00
			17.333	BV	3340.51	32.47
			18.028	VV m	1332.92	12.96
			18.526	BV	1181.37	11.48
				总和	10288.05	

单次进样报告

Agilent Technologies

数据文件:	11616.A Glu. acid20200612 112520.dx		
序列名称:	6696.A profile 3	项目名称:	脂肪酸
样品名称:	11616.A Glu. acid	操作者:	系统
仪器:	7890B	进样日期:	2020-06-12 11:29:39+08:00
进样体积:	1.000	位置:	103
采集方法:	脂肪酸测定方法25min.amx	类型:	样品
处理方法:	GC_LC<br>面积百分比_DefaultMethod.pmx	样品含量:	0.00
手动修改:	手动积分		

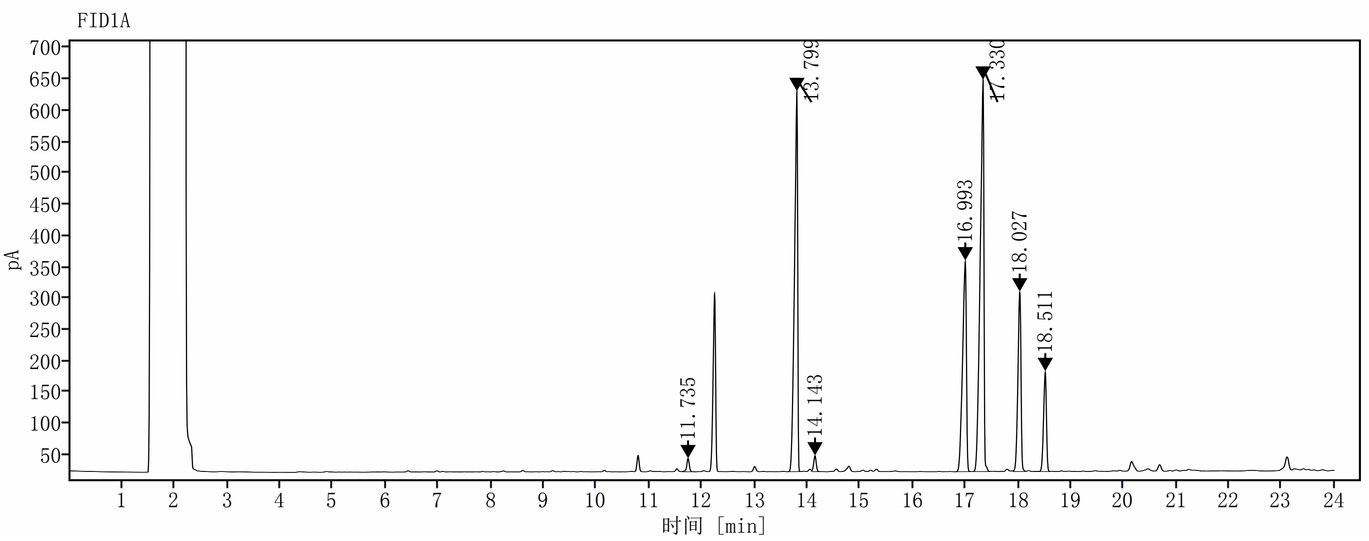

信号: FID1A

名称	化合物 浓度	化合物 含量	保留时间 [min]	类型	峰面积	峰面积%
			11.735	VB	72.41	0.79
			13.799	BB	2524.20	27.44
			14.143	VB	84.35	0.92
			16.993	BB	1686.59	18.34
			17.330	BV	3069.67	33.38
			18.027	VV	1160.24	12.61
			18.511	BV	599.94	6.52
				总和	9197.40	

数据文件:	11616.A Methionine20200612 115340.dx		
序列名称:	6696.A profile 3	项目名称:	脂肪酸
样品名称:	11616.A Methionine	操作者:	系统
仪器:	7890B	进样日期:	2020-06-12 11:58:03+08:00
进样体积:	1.000	位置:	104
采集方法:	脂肪酸测定方法25min.amx	类型:	样品
处理方法:	GC_LC<br>面积百分比_DefaultMethod.pmx	样品含量:	0.00
手动修改:	手动积分		

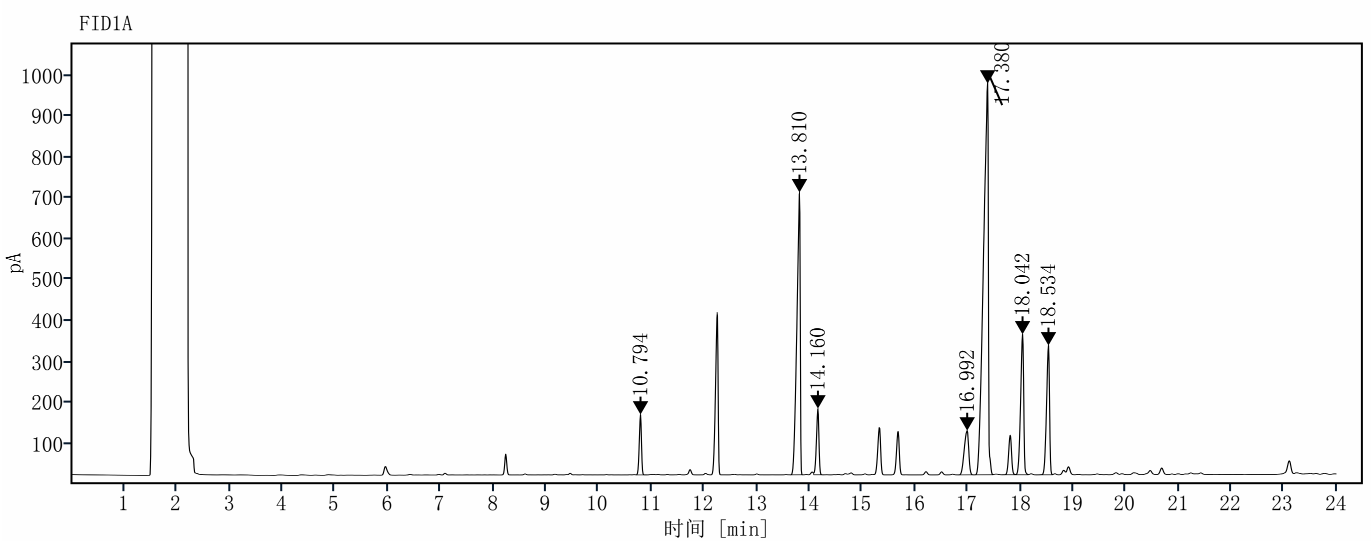

信号: FID1A

名称	化合物 浓度	化合物 含量	保留时间 [min]	类型	峰面积	峰面积%
			10.794	VB	424.50	3.08
			13.810	VB	3025.31	21.96
			14.160	VB	524.02	3.80
			16.992	BB	636.22	4.62
			17.380	BV	6145.21	44.61
			18.042	BV m	1767.94	12.83
			18.534	BV	1251.48	9.09
				总和	13774.67	

数据文件:	11616.A Glycine20200612 122205.dx		
序列名称:	6696.A profile 3	项目名称:	脂肪酸
样品名称:	11616.A Glycine	操作者:	系统
仪器:	7890B	进样日期:	2020-06-12 12:26:29+08:00
进样体积:	1.000	位置:	105
采集方法:	脂肪酸测定方法25min.amx	类型:	样品
处理方法:	GC_LC<br>面积百分比_DefaultMethod.pmx	样品含量:	0.00
手动修改:	手动积分		

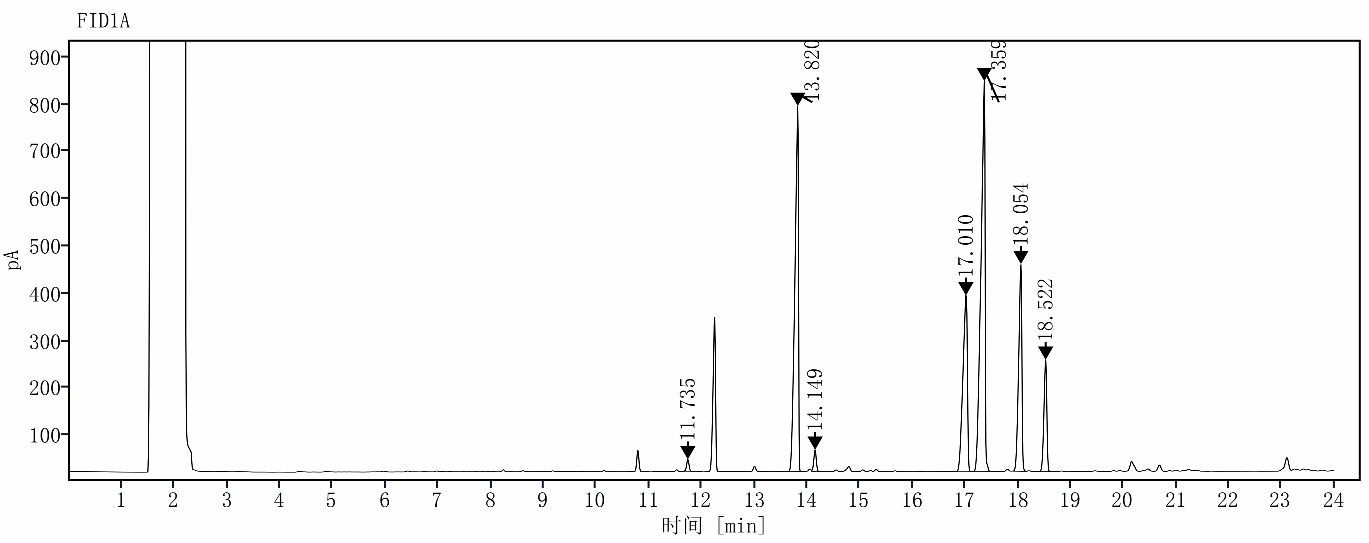

信号: FID1A

名称	化合物 浓度	化合物 含量	保留时间 [min]	类型	峰面积	峰面积%
			11.735	VB	88.05	0.67
			13.820	VB	3586.54	27.16
			14.149	VB	149.85	1.13
			17.010	BB	2185.11	16.55
			17.359	BV	4452.73	33.72
			18.054	VV	1845.24	13.97
			18.522	BV	898.46	6.80
				总和	13205.99	

单次进样报告

Agilent Technologies

**数据文件:**	11616.A Leucine20200612 125030.dx		
**序列名称:**	6696.A profile 3	**项目名称:**	脂肪酸
**样品名称:**	11616.A Leucine	**操作者:**	系统
**仪器:**	7890B	**进样日期:**	2020-06-12 12:54:55+08:00
**进样体积:**	1.000	**位置:**	106
**采集方法:**	脂肪酸测定方法25min.amx	**类型:**	样品
**处理方法:**	GC_LC<br>面积百分比_DefaultMethod.pmx	**样品含量:**	0.00
**手动修改:**	手动积分		

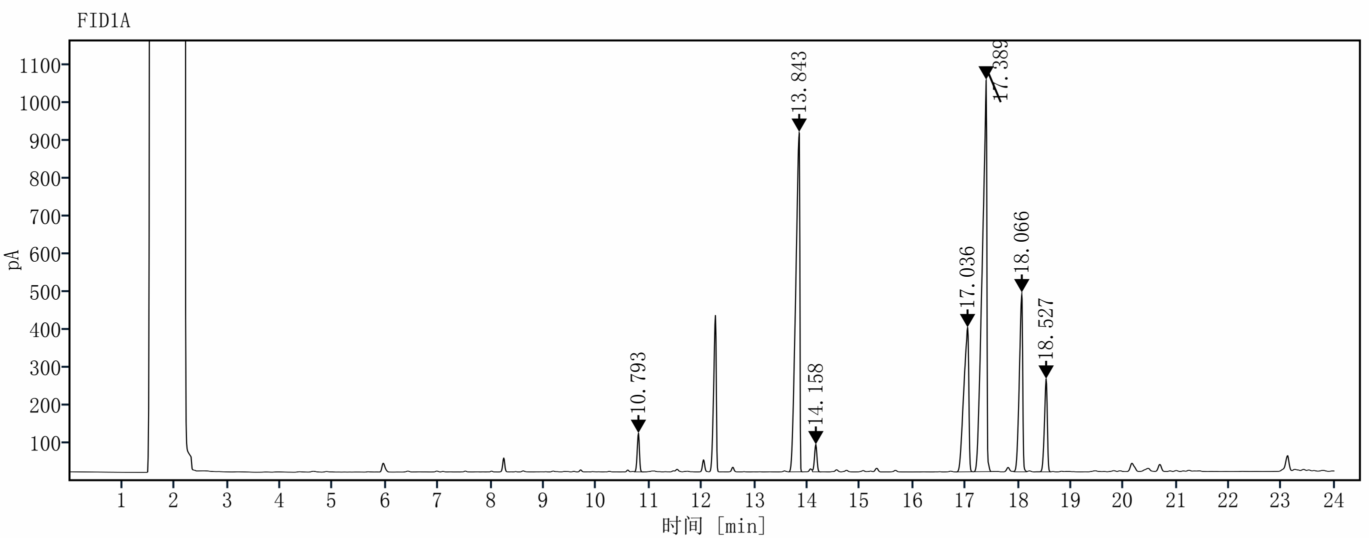

信号: FID1A

名称	化合物 浓度	化合物 含量	保留时间 [min]	类型	峰面积	峰面积%
			10.793	VB	298.28	1.75
			13.843	VB	4804.67	28.26
			14.158	VB	235.12	1.38
			17.036	BB	2501.24	14.71
			17.389	BB	6159.59	36.23
			18.066	VV	2070.28	12.18
			18.527	BV	931.45	5.48
				总和	17000.63	

单次进样报告

数据文件:	11616.A Lycine20200612 131858.dx		
序列名称:	6696.A profile 3	项目名称:	脂肪酸
样品名称:	11616.A Lycine	操作者:	系统
仪器:	7890B	进样日期:	2020-06-12 13:23:19+08:00
进样体积:	1.000	位置:	107
采集方法:	脂肪酸测定方法25min.amx	类型:	样品
处理方法:	GC_LC 面积百分比_DefaultMethod.pmx	样品含量:	0.00
手动修改:	手动积分		

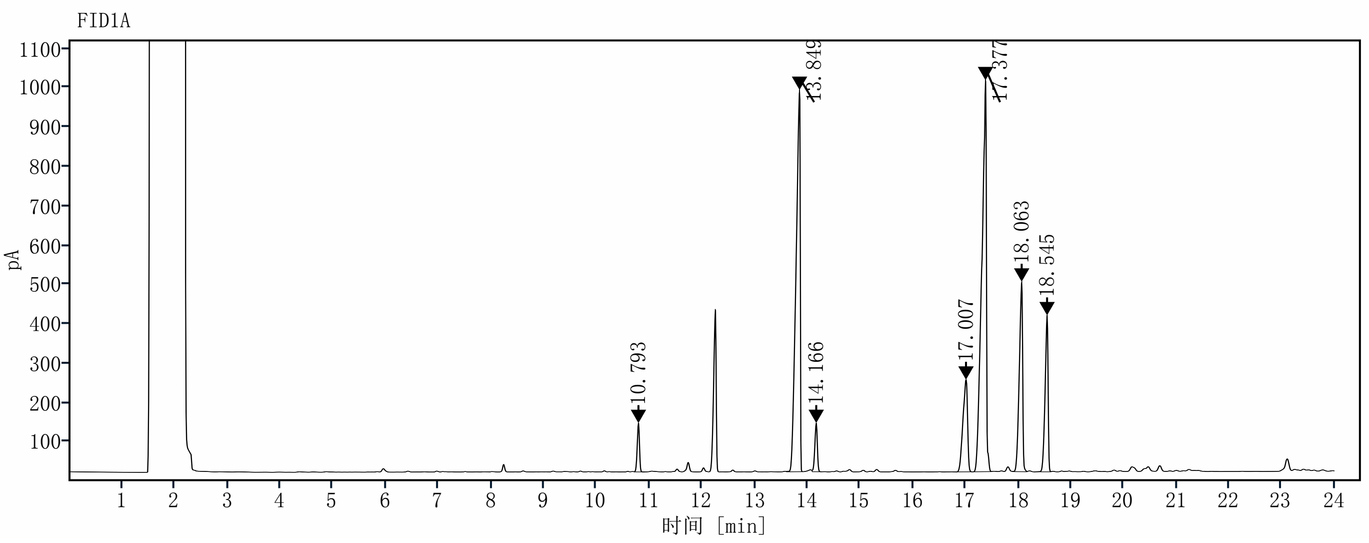

信号: FID1A

名称	化合物 浓度	化合物 含量	保留时间 [min]	类型	峰面积	峰面积%
			10.793	VB	367.61	2.14
			13.849	VB	5332.36	31.00
			14.166	VB	405.42	2.36
			17.007	BB	1413.12	8.22
			17.377	BV	6006.90	34.92
			18.063	VV	2119.95	12.32
			18.545	BV	1555.42	9.04
				总和	17200.78	

单次进样报告

**数据文件:**	11616.A Iso-leucine20200612 134720.dx		
**序列名称:**	6696.A profile 3	**项目名称:**	脂肪酸
**样品名称:**	11616.A Iso-leucine	**操作者:**	系统
**仪器:**	7890B	**进样日期:**	2020-06-12 13:51:45+08:00
**进样体积:**	1.000	**位置:**	108
**采集方法:**	脂肪酸测定方法25min.amx	**类型:**	样品
**处理方法:**	GC_LC<br>面积百分比_DefaultMethod.pmx	**样品含量:**	0.00
**手动修改:**	手动积分		

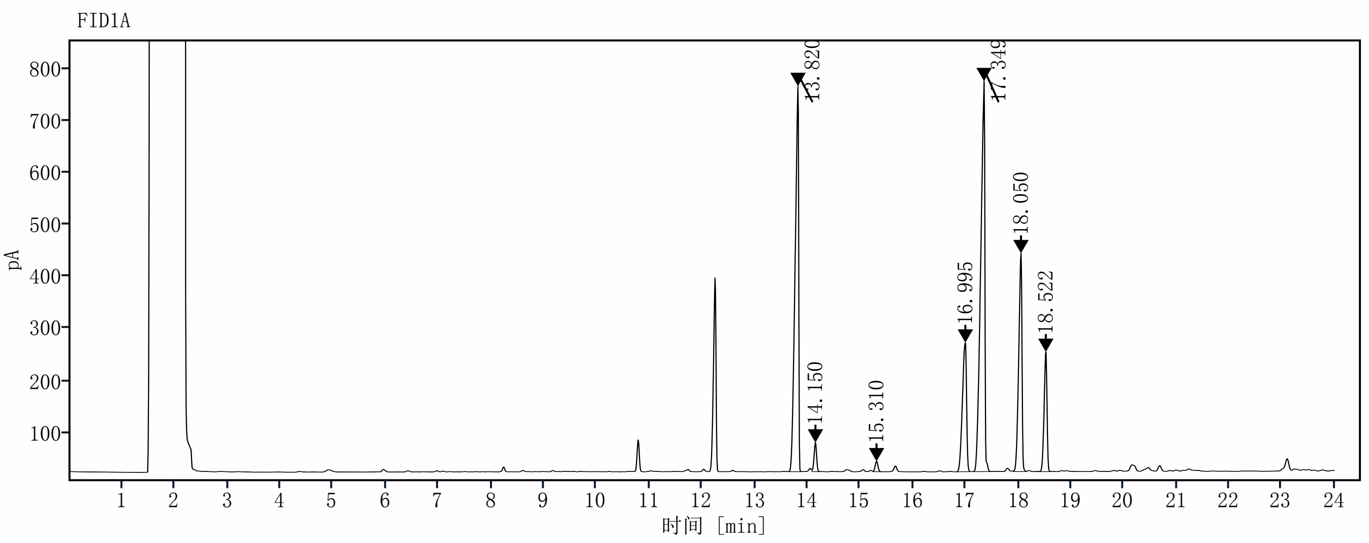

信号: FID1A

名称	化合物 浓度	化合物 含量	保留时间 [min]	类型	峰面积	峰面积%
			13.820	VV	3458.33	29.30
			14.150	VB	184.38	1.56
			15.310	VB	74.04	0.63
			16.995	BB	1347.38	11.42
			17.349	BV	4072.73	34.51
			18.050	VV	1786.02	15.13
			18.522	BV	879.91	7.46
				总和	11802.80	

单次进样报告

Agilent Technologies

数据文件:	11616.A Aspargine20200612 141547.dx		
序列名称:	6696.A profile 3	项目名称:	脂肪酸
样品名称:	11616.A Aspargine	操作者:	系统
仪器:	7890B	进样日期:	2020-06-12 14:20:15+08:00
进样体积:	1.000	位置:	109
采集方法:	脂肪酸测定方法25min.amx	类型:	样品
处理方法:	GC_LC<br>面积百分比_DefaultMethod.pmx	样品含量:	0.00
手动修改:	手动积分		

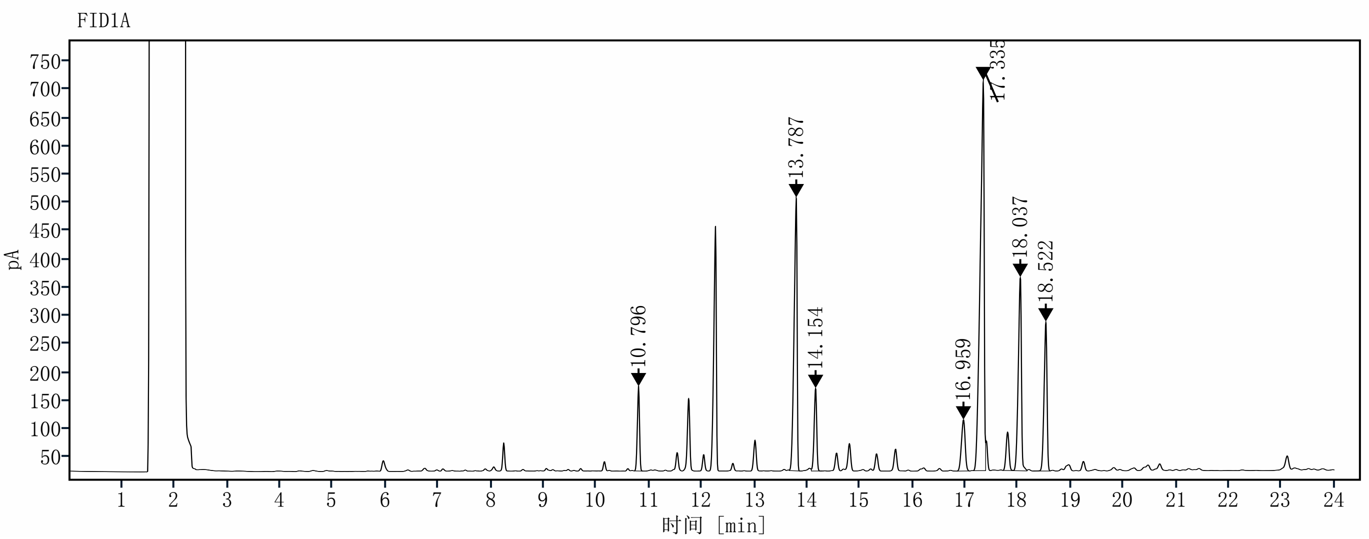

信号: FID1A

名称	化合物 浓度	化合物 含量	保留时间 [min]	类型	峰面积	峰面积%
			10.796	BB	429.41	4.49
			13.787	VB	1908.26	19.94
			14.154	VB	494.59	5.17
			16.959	BB	443.81	4.64
			17.335	BV	3599.29	37.60
			18.037	BV m	1676.49	17.51
			18.522	BV	1020.03	10.66
				总和	9571.88	

单次进样报告

Agilent Technologies

数据文件:	11616.A Proline20200612 144417.dx		
序列名称:	6696.A profile 3	项目名称:	脂肪酸
样品名称:	11616.A Proline	操作者:	系统
仪器:	7890B	进样日期:	2020-06-12 14:48:41+08:00
进样体积:	1.000	位置:	110
采集方法:	脂肪酸测定方法25min.amx	类型:	样品
处理方法:	GC_LC<br>面积百分比_DefaultMethod.pmx	样品含量:	0.00
手动修改:	手动积分		

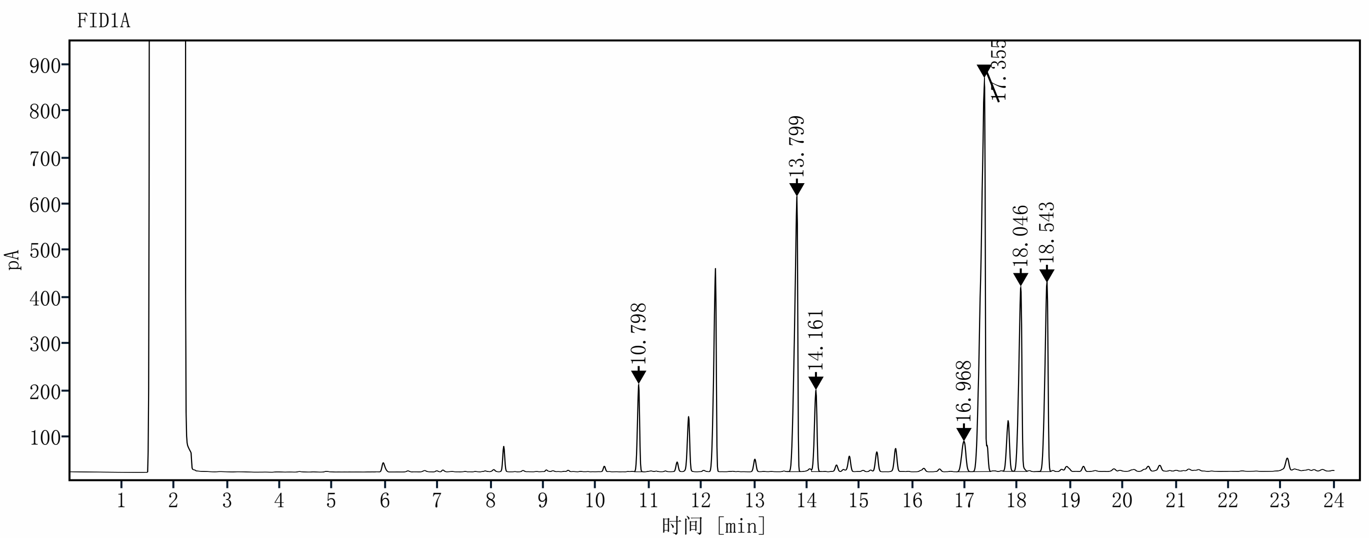

信号: FID1A

名称	化合物 浓度	化合物 含量	保留时间 [min]	类型	峰面积	峰面积%
			10.798	VB	547.53	4.33
			13.799	VB	2457.99	19.44
			14.161	VB	600.11	4.75
			16.968	BB	363.04	2.87
			17.355	BV	4918.84	38.90
			18.046	VV m	2068.38	16.36
			18.543	BV	1688.74	13.36
				总和	12644.63	

单次进样报告

数据文件:	11616.A Control20200612 151244.dx		
序列名称:	6696.A profile 3	项目名称:	脂肪酸
样品名称:	11616.A Control	操作者:	系统
仪器:	7890B	进样日期:	2020-06-12 15:17:07+08:00
进样体积:	1.000	位置:	111
采集方法:	脂肪酸测定方法25min.amx	类型:	样品
处理方法:	GC_LC<br>面积百分比_DefaultMethod.pmx	样品含量:	0.00
手动修改:	手动积分		

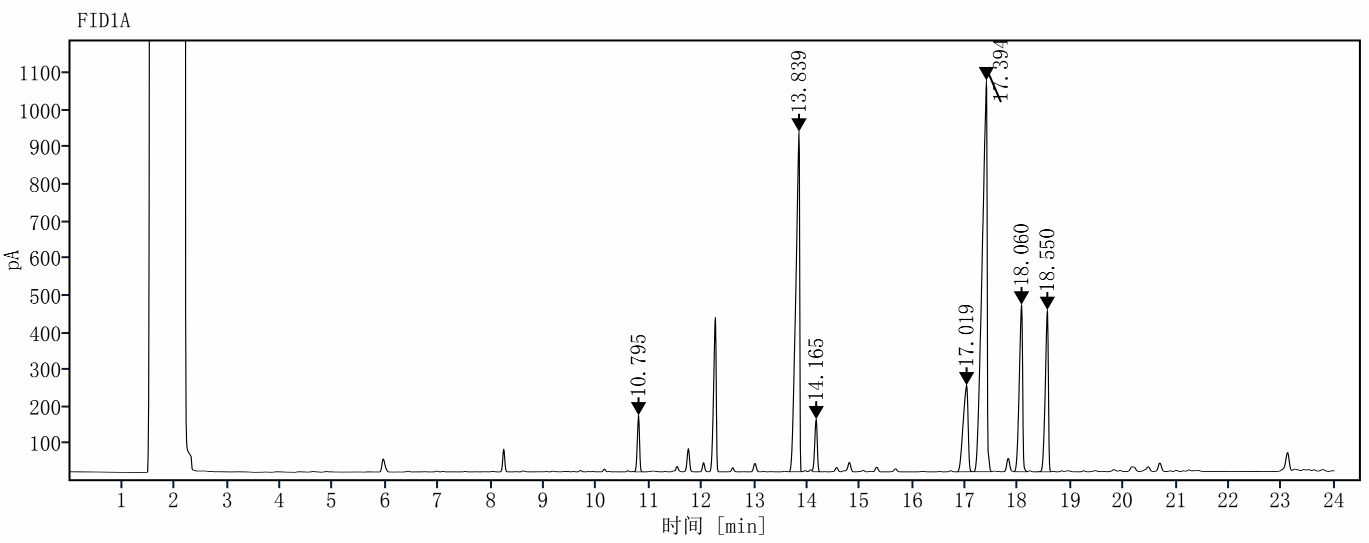

信号: FID1A

名称	化合物 浓度	化合物 含量	保留时间 [min]	类型	峰面积	峰面积%
			10.795	VB	444.92	2.52
			13.839	VB	4654.39	26.33
			14.165	VB	469.72	2.66
			17.019	BB	1498.75	8.48
			17.394	BV	6858.49	38.80
			18.060	VV	1985.22	11.23
			18.550	BV	1764.44	9.98
				总和	17675.94	
